# Analysis of human ES cell differentiation establishes that the dominant isoforms of the lncRNAs *RMST* and *FIRRE* are circular

**DOI:** 10.1186/s12864-018-4660-7

**Published:** 2018-04-20

**Authors:** Osagie G. Izuogu, Abd A. Alhasan, Carla Mellough, Joseph Collin, Richard Gallon, Jonathon Hyslop, Francesco K. Mastrorosa, Ingrid Ehrmann, Majlinda Lako, David J. Elliott, Mauro Santibanez-Koref, Michael S. Jackson

**Affiliations:** 1 Institute of Genetic Medicine, Newcastle University, International Centre for Life, Central Parkway, Newcastle upon Tyne, NE1 3BZ UK; 2 Present Address: The European Bioinformatics Institute (EMBL-EBI), Wellcome Genome Campus, Cambridge, CB10 1SD UK; 3 Present Address: Institute of Cellular Medicine, William Leech Building, Medical School, Newcastle University, Newcastle-upon-Tyne, NE2 4HH UK; 4 Present Address: Lions Eye Institute, 2 Verdun Street, Nedlands, WA 6009 Australia

## Abstract

**Background:**

Circular RNAs (circRNAs) are predominantly derived from protein coding genes, and some can act as microRNA sponges or transcriptional regulators. Changes in circRNA levels have been identified during human development which may be functionally important, but lineage-specific analyses are currently lacking. To address this, we performed RNAseq analysis of human embryonic stem (ES) cells differentiated for 90 days towards 3D laminated retina.

**Results:**

A transcriptome-wide increase in circRNA expression, size, and exon count was observed, with circRNA levels reaching a plateau by day 45. Parallel statistical analyses, controlling for sample and locus specific effects, identified 239 circRNAs with expression changes distinct from the transcriptome-wide pattern, but these all also increased in abundance over time. Surprisingly, circRNAs derived from long non-coding RNAs (lncRNAs) were found to account for a significantly larger proportion of transcripts from their loci of origin than circRNAs from coding genes. The most abundant, circ*RMST*:E12-E6, showed a > 100X increase during differentiation accompanied by an isoform switch, and accounts for > 99% of *RMST* transcripts in many adult tissues. The second most abundant, circ*FIRRE*:E10-E5, accounts for > 98% of *FIRRE* transcripts in differentiating human ES cells, and is one of 39 *FIRRE* circRNAs, many of which include multiple unannotated exons.

**Conclusions:**

Our results suggest that during human ES cell differentiation, changes in circRNA levels are primarily globally controlled. They also suggest that *RMST* and *FIRRE*, genes with established roles in neurogenesis and topological organisation of chromosomal domains respectively, are processed as circular lncRNAs with only minor linear species.

**Electronic supplementary material:**

The online version of this article (10.1186/s12864-018-4660-7) contains supplementary material, which is available to authorized users.

## Background

Circular RNAs (circRNAs) are a numerically abundant RNA species, generally expressed at low levels relative to mRNAs [1, 2], which can be defined in silico by the presence of “shuffled” or “back-spliced” exons in an order inconsistent with genomic DNA. Rare trans-splicing events between different pre-mRNA molecules can, however, also generate rearranged exon junctions [3, 4], meaning that additional evidence such as resistance to RNase R [5], migration assays [6], or comparison of read depth inside and outside of the circRNA [7], is required to confirm circularity in individual cases.

CircRNA biogenesis can be promoted by intronic homology [5, 8, 9], and knock-down of the dsRNA editing enzyme ADAR1 [10, 11], or the RNA helicase DHX9 which can interact with ADAR1 [12], leads to upregulation of circRNA expression. Cassette exons have been shown to be over-represented within circRNAs [13], and a combination of RNAi screens and specific gene analyses have established that RNA binding proteins including MBL [14], QKI [15], and heterogeneous nuclear ribonuncleoproteins (hnRNPs) [16] promote formation of some circRNAs. Furthermore, both depletion of pre-mRNA splicing factors, and transcript termination read-through, can increase circRNA levels relative to linear [17].

The vast majority of circRNAs are derived from protein coding genes and have no known function [18, 19], but there is evidence that some can act as micro RNA sponges [20–23], some can enhance the activity of their own promoters [24], some can contribute to the proteome [25–27], and a growing number are being associated with disease (reviewed in [28]). For example, a circRNA isoform of the long non-coding RNA (lncRNA) *ANRIL1* has been implicated in atheroprotection [29, 30], while circRNAs created by an oncogenic fusion have been reported to influence tumour progression [31].

Given the evidence for biological impact, changes in circRNA expression are of interest as they could be indicative of function. CircRNAs are almost always co-expressed with linear transcripts from the same loci [32], and their expression has been shown to increase relative to linear RNA during development [11], proliferation [33], and ageing [34, 35]. Global increases over time have been observed in heart and lung, but are most pronounced in neural tissues [11, 34–37]. Moreover, divergent expression patterns of linear and circular RNAs from the same loci have been highlighted in multiple neuronal tissues, at a variety of developmental stages and in different species [11, 36, 38], providing circumstantial evidence for independent regulation of linear and circular isoforms.

The circRNA population of human ES cells during early differentiation has also been investigated and Circ*Birc6* [39], together with a linear trans-spliced RNA derived from the lncRNA *RMST* (ts*RMST* [40]), have been reported to play roles in maintenance of pluripotency [40]. However, analyses of long-term differentiation series, to investigate changes in circRNA abundance within defined cell lineages, are currently lacking. Here, we have differentiated human ES cells (H9 line) towards 3D laminated retina using an established 90 day protocol [41, 42], and have performed total RNAseq to a depth of > 100 million reads in 3 biological replicates at 3 time-points (0, 45, 90 days). Global circRNA levels increased dramatically between day 0 and day 45, and circRNA size and exon number also increased. When we controlled both for this transcriptome-wide increase, and for locus specific changes in total expression, we identified 239 circRNAs with significantly altered expression levels between time-points. All also increased in abundance, consistent with circRNA levels being controlled globally during differentiation. Surprisingly, however, we identified a significant difference in circRNA levels between coding and non-coding genes, and found evidence that two lncRNAs with known functions in early development, *RMST* and *FIRRE*, may warrant classification as circular lncRNAs in human.

## Results

**Fig. 1:**
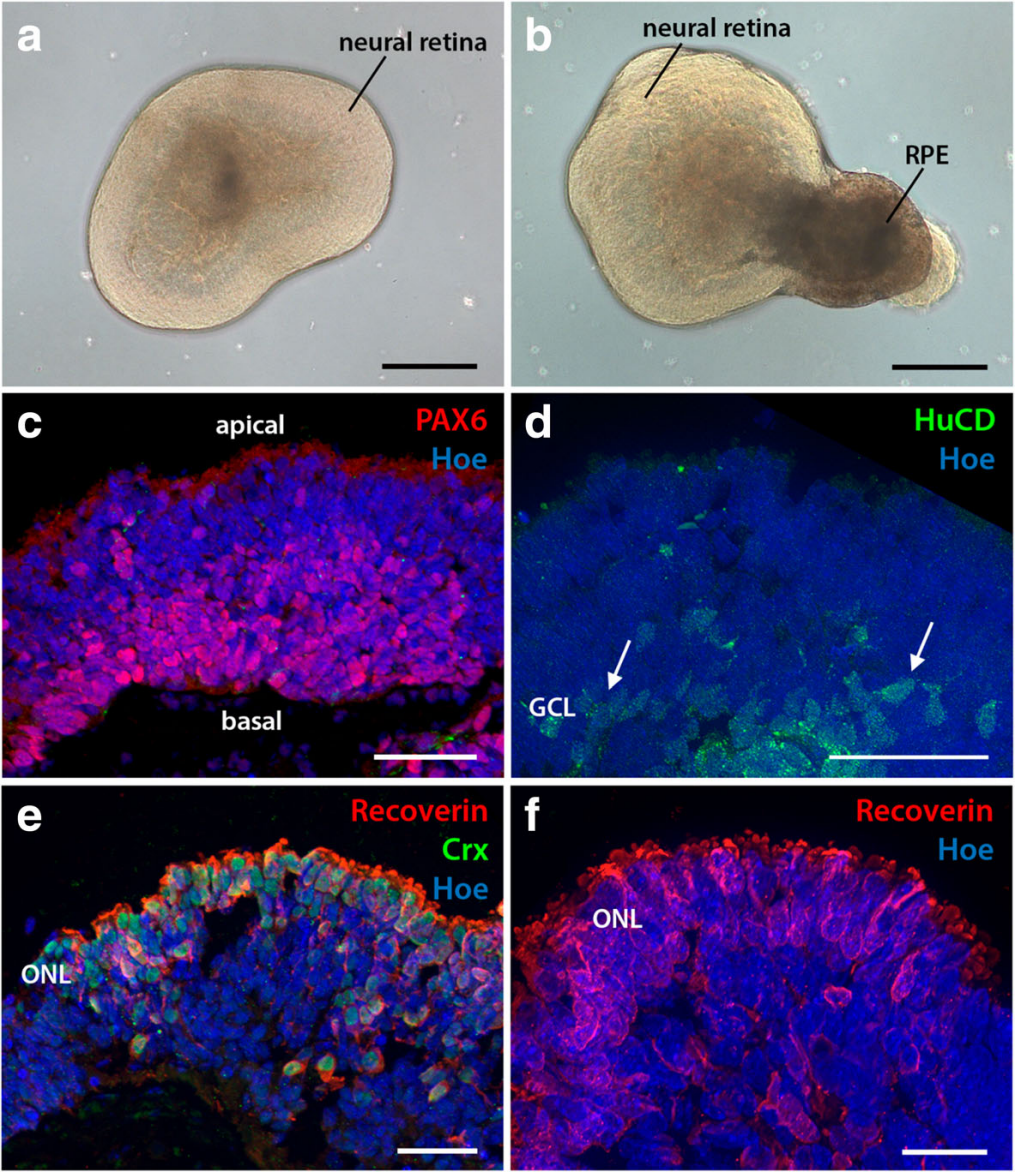
Neuronal differentiation in retinal organoids. **a**-**b** Retinal organoids on day 23 of differentiation displaying (**a**) phase bright neural retinal tissue and (**b**) retinal pigmented epithelium (RPE). **c**-**f** Immunostained cryosections of retinal organoids on day 90 of differentiation counterstained with Hoechst demonstrating the laminar organisation of retinal tissue and presence of (**c**) Pax6+ retinal progenitors, inner retinal neurons and (**d**) HuC/D+ retinal ganglion cells at the basal aspect, and (**e**, **f**) outer retinal phenotypes including Crx + and recoverin+ photoreceptors towards the apical surface. GCL = ganglion cell layer, ONL = outer nuclear layer. Scale bars; a, b = 200 μm; c, d = 50 μm; e, f = 20 μm

To identify circRNAs potentially involved in the development of a defined cell lineage, we differentiated triplicate human ES cell cultures towards a retinal phenotype [41], with and without the presence of IGF-1 which has been reported to enhance retinal differentiation [42]. Embryoid bodies (EBs) with retinal features developed in all replicates (see Methods), and immunostained cryosections of organoids at day 90 confirmed the presence of laminar retinal tissue organisation, with Pax6 positive retinal progenitors, HuC/D positive retinal ganglion cells, and Crx/Recoverin positive photoreceptors (Fig. 1).

To assess gene expression, total RNA was extracted at day 0, 45 and 90, ribodepleted, and sequenced to a depth of over 100 million reads per replicate (see Methods and Additional file 1). Consistent with the phenotypic changes observed, gene ontologies relevant to the eye were significantly over-represented among genes differentially expressed between time points (e.g. phototransduction, sensory perception of light stimulus, and structural constituent of the eye lens, Additional file 2), while known pluripotency genes and eye field markers were downregulated and upregulated respectively (Additional file 3: Figure S1). In contrast, the impact of IGF-1 upon transcription was very specific, with 30 genes differentially expressed between treated and untreated samples, many of which are implicated in development or neuronal function (See Additional file 2). Of particular interest are *WNT2B* and *FGF16*, as inhibition of Wnt signalling has been shown to enhance forebrain patterning [41] and FGF signalling modulates retinal progenitor proliferation and fate [43], and *IRS4* and *LIN28A*, both of which are already known to be involved in IGF-1 signalling [44, 45]. Thus, while few genes are affected by IGF-1, they include logical candidates for involvement in the reported impact of this mitogen [42].

### circRNA levels increase dramatically during the first 45 days of differentiation

**Fig. 2:**
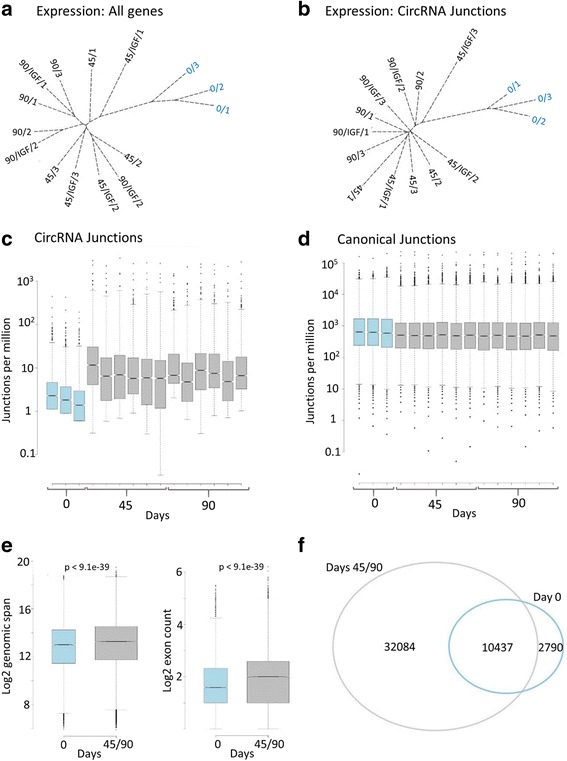
circRNA expression increases upon differentiation. **a** Phylogram based on expression levels of all genes. **b** Phylogram based on circRNA (back-splice) junction counts. Both generated using hierarchical clustering of Euclidian distances. **c**-**d** Box and whisker plots of circRNA and canonical junction counts from all genes which generate circRNAs. CircRNAs increase from ~ 0.3 to 1-2% of all junctions between day 0 and 45. **e** Box and whisker plots showing (left) genomic span of circRNAs in day 0 (*n* = 13,146) versus differentiated (day 45 and 90, *n* = 42,521) untreated samples, and (right) exon counts of circRNAs in day 0 only (*n* = 2790) versus differentiated only (*n* = 32,084) untreated samples. For exon count calculations, all GENCODE exons between circRNA donor and acceptor exons were included. For all box-plots, medians and upper and lower quartiles are shown, with outliers as solid circles. **f** Venn diagram showing distribution of circRNA junctions identified in untreated day 0 samples, relative to differentiated samples (days 45 and 90). Comparable results were obtained with IGF-1 treated samples

We used PTESFinder [46], a software tool with high sensitivity and specificity [47], to quantify all circRNA (back-splice) and canonical junctions at all time-points (see Methods and Fig. 2). A total of 58,528 distinct circRNAs were identified, the majority of which were also found using other circRNA identification tools ([8, 20, 34], see Additional file 3: Figure S2). Hierarchical clustering using normalised gene expression values (Fig. 2a), circRNA junction counts (Fig. 2b), or canonical exon junction counts (not shown), all identified day 0 samples as a distinct group. This distinction is clear from a heat map of genes differentially expressed between time-points and treatments (Additional file 3: Figure S3), and is consistent with the limited expression signature associated with IGF-1 treatment. The limited impact on circRNA levels is also consistent with Enuka et al. [32] who found that stimulation of mammary cells by EGF did not affect circRNA abundance.

Relative to canonical junctions, circRNA junctions were present at low levels at all time-points (Fig. 2c-d), but they showed a significant ~ 4-5X increase between day 0 and day 45 (*p* = 0.0044 Wilcoxon rank sum test, Fig. 2c). Significant increases were also observed in the physical distance between circRNA donor and acceptor exons (genomic span), and the number of exons circRNAs contain (Fig. 2e, both *p* < 9.2e-39 Wilcoxon rank sum test). Approximately 6% of the circRNAs identified were found only in undifferentiated samples (day 0, Fig. 2f), compared to over 70% found only in differentiated samples (Day 45/90), and detailed analysis found no evidence of abundant circRNAs specific to undifferentiated ES cells (Additional file 3: Figure S4).

**Fig. 3:**
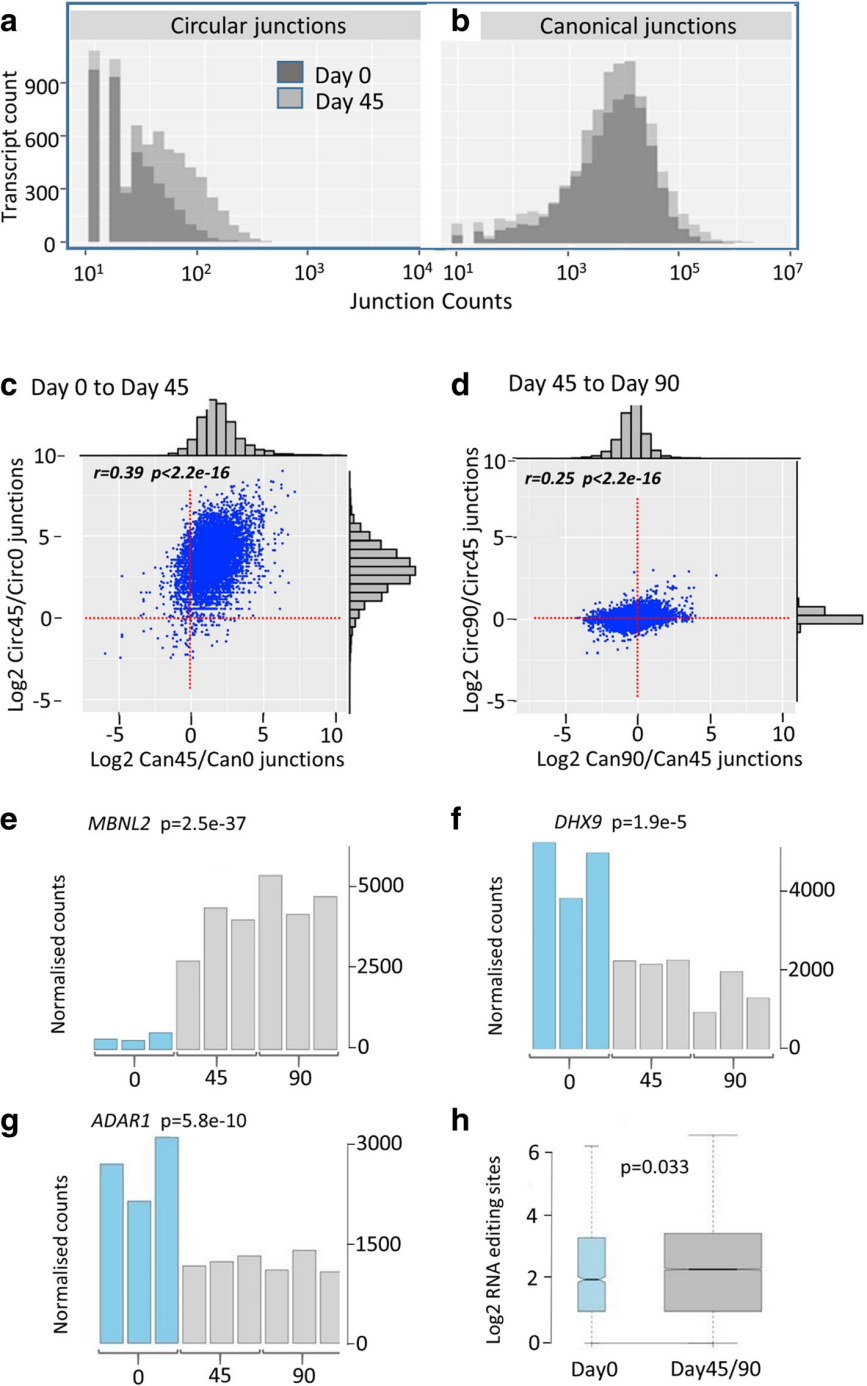
circRNA expression reaches a plateau by day 45. **a**-**b** Histograms comparing junction counts per transcript in day 0 and day 45. **c-d** Comparison of changes in back-splice and canonical junction count frequencies between time points. Junction counts were normalised to total read counts at each time-point. Distributions are shown on both axes, together with correlation coefficients and associated *p*-values. **e**-**g** Expression of genes implicated in circRNA biogenesis in untreated samples*.* Associated p-values from t-tests comparing means in day 0 versus day 45/90 are shown. **h** Box and whisker plot comparing frequencies of RNA editing sites found within 1000 bp windows flanking circRNA junctions in undifferentiated (day 0) or differentiated (day 45 and day 90) samples only, with associated p-value (Wilcoxon rank-sum test)

To investigate the increase in circRNAs in detail, junction count distributions from all genes were then examined at day 0 and day 45. This established that the number of both circRNAs and circRNA junctions increased more dramatically between day 0 and day 45 (Fig. 3a) than the number of independent transcripts and canonical junctions (Fig. 3b). To assess these changes at the gene level, ratios of normalised circRNA junction counts at day 45 relative to day 0 for each gene were plotted against the corresponding ratios for canonical junctions (Fig. 3c): Both circRNA and canonical junctions increased between day 0 and day 45, but the increase in circRNA junctions was more pronounced (median increase ~8X, log_2_ = 3). In contrast, there was little change in circRNA or canonical junction abundance between days 45 and 90, with circRNA levels remaining particularly stable (Fig. 3d). Despite this, ratios were correlated in both comparisons, consistent with circRNA levels at each locus being linked to total transcription.

As Muscleblind [14] and QKI [15] RNA binding proteins can promote circularisation, their expression was investigated. Although *QKI* remained unchanged (data not shown), 2 out of the 3 human Muscleblind genes showed a significant increase by day 45 (Fig. 3e and Additional file 3: Figure S1). Furthermore, the levels of *ADAR1* [10, 11] and *DHX9* [12], which can reduce circRNA levels, decreased significantly (Fig. 3f-g), consistent with involvement in the observed circRNA upregulation. A small but significant excess of RNA editing sites (targeted by *ADAR1*) was also observed within 1000 bp of circRNA junctions found only in differentiated time-points, when compared to junctions found only at day 0 (*p* = 0.0329 Wilcoxon rank sum test, Fig. 3h).

### CircRNA expression change is defined by global upregulation and locus specific transcription levels

The upregulation of circRNAs prior to day 45, and their subsequent relative stability, suggests that their abundance is controlled at the transcriptome level during human ES cell differentiation. To identify circRNAs showing differential expression (DE) patterns, independent both of the global increase and of changes in total expression at their loci of origin, we performed two sets of pairwise comparisons between time-points: One (sample-level DE analysis) controlled for differences in library size and total circRNA levels between samples, the other (locus-level DE analysis) controlled for locus specific changes in total gene expression. Fisher exact tests were first performed, using junction counts summed across replicates, to identify candidate differentially expressed (DE) circRNAs. Then, to mitigate against sample heterogeneity, read counts of these candidate DE circRNAs in each replicate were re-analysed using two-tailed t-tests (see methods and Additional file 3: Figure S5).

**Fig. 4:**
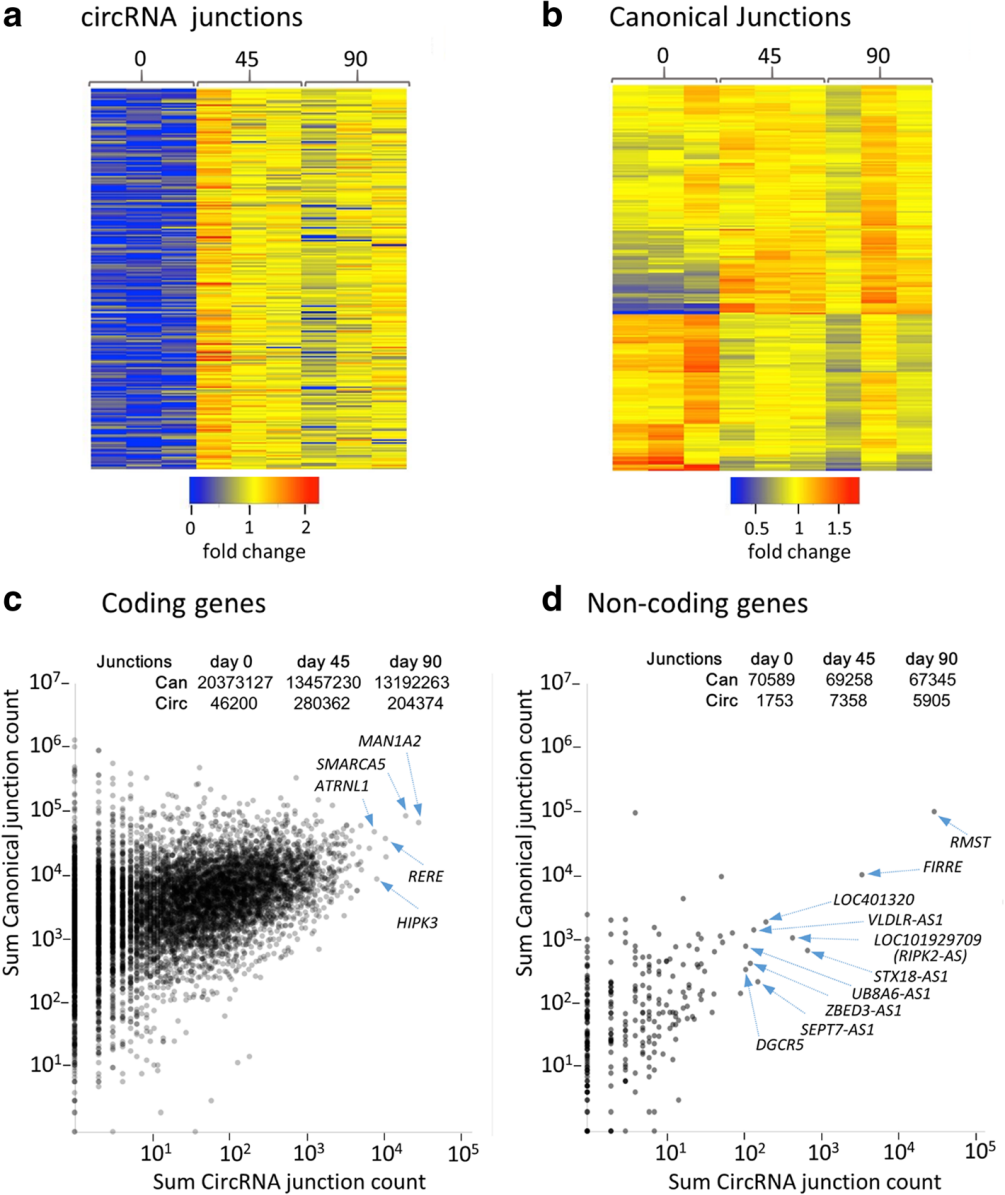
Expression increase of DE genes and abundance with respect to coding capacity. **a**-**b** Expression heat maps showing relative frequency of untreated sample junction counts from 239 DE transcripts identified in both sample-level and locus-level t-tests (see methods). **c** Total canonical junctions versus total circRNA junctions in all circRNA producing coding genes (summed across all samples). The 5 genes with the highest circRNA counts are indicated. **d** Total canonical junctions versus circRNA junctions in all circRNA producing non-coding genes (summed across all samples). The 10 genes with the highest circRNA counts are indicated. Total numbers of canonical and circRNA junctions in untreated samples at each time-point are also shown, and confirm that the increase in junction counts between days 0 and 45 affects circRNAs from both coding and non-coding genes

In pairwise comparisons between the day 45 and day 90 samples (rows 5-8 Table 1) between 1 and 256 DE circRNAs were identified using Fisher’s exact tests, but only 1 was significant in either of the subsequent t-tests. In contrast, over 2500 transcripts were identified in each pairwise comparison between day 0 samples and later time-points, and 46-496 were significant in the subsequent t-tests (rows 1-4 Table 1). However, only 239 (~ 0.6% of all circRNAs analysed) gave 1 or more significant results in both analyses (Table 1 and Additional file 4). Expression heat maps illustrate that circRNA junction levels of all 239 increase upon differentiation (Fig. 4a), with more modest changes in canonical junction counts (Fig. 4b). Thus, these circRNAs differ from the broader population only in the magnitude/consistency of expression change, not direction. Gene Ontology analysis did identify enrichment of some molecular processes and biological functions among genes from which these circRNAs are generated (Additional file 5), but none are specifically relevant to differentiation or neuronal/retinal development. In an effort to increase power, this analysis was repeated with day 45 and day 90 samples combined. As expected, this produced a larger number of DE transcripts (> 2000), but the direction of change in circRNA levels remained uniform (Additional file 3: Figure S6), providing no evidence for regulation of specific circRNAs independent of the global upregulation observed prior to day 45.

**Table 1:** Numbers of DE transcripts identified in pairwise time-point analyses, controlling for transcriptome wide changes in circRNA levels and locus specific changes in total expression

Comparison	Locus-level tests		Sample-level tests		Identified by both t-tests
	Fisher	t	Fisher	t	
day 0 v day 45	4207	63	6411	60	8
day 0 v day 90	2671	80	4577	46	12
day 0 v day 45 IGF-1	2965	496	4395	496	197
day 0 v day 90 IGF-1	2786	126	4362	148	29
day 45 v day 45 IGF-1	60	0	256	0	0
day 45 v day 90	121	0	208	0	0
day45 IGF-1 v day 90 IGF-1	10	1	56	0	0
day 90 v day 90 IGF-1	1	0	15	0	0

### Non-coding circRNA producing genes have higher circular/linear ratios than coding genes

The vast majority of the 239 significant DE circRNAs have been identified previously and are derived from coding genes (Additional file 3: Figure S4). However, the most abundant is from *RMST*, a lncRNA known to be involved in the regulation of neural stem cell fate [48]. As the functional relationship between linear and circular transcripts from coding and non-coding loci will be different, we analysed the relationship between circRNA and canonical junction frequency with respect to coding potential (Fig. 4c-d). This established that circRNAs from non-coding genes make up a higher proportion of the total transcripts from their loci of origin than circRNAs from coding genes (*p* < 1 × 10^− 5^ Wilcoxon rank-sum test), with a mean circular to linear junction ratio of 0.25 (IQR 0.18) compared to 0.04 (IQR 0.024) for coding genes, despite non-coding genes being generally expressed at lower levels. Interestingly, the 2nd most abundant non-coding circRNA was from the lncRNA *FIRRE*, implicated in both adipogenesis in mouse [49] and chromosomal nuclear localisation [50, 51], with anti-sense RNAs of unknown function also having a high proportion of circRNA junctions. Because of the known involvement of both *RMST* and *FIRRE* in developmental processes, we analysed read depth and splice junction frequency in these two genes in detail.

### CircRMST:E12-E6 can account for > 99% of *RMST* transcription, and upregulation is consistent with a promoter change

**Fig. 5:**
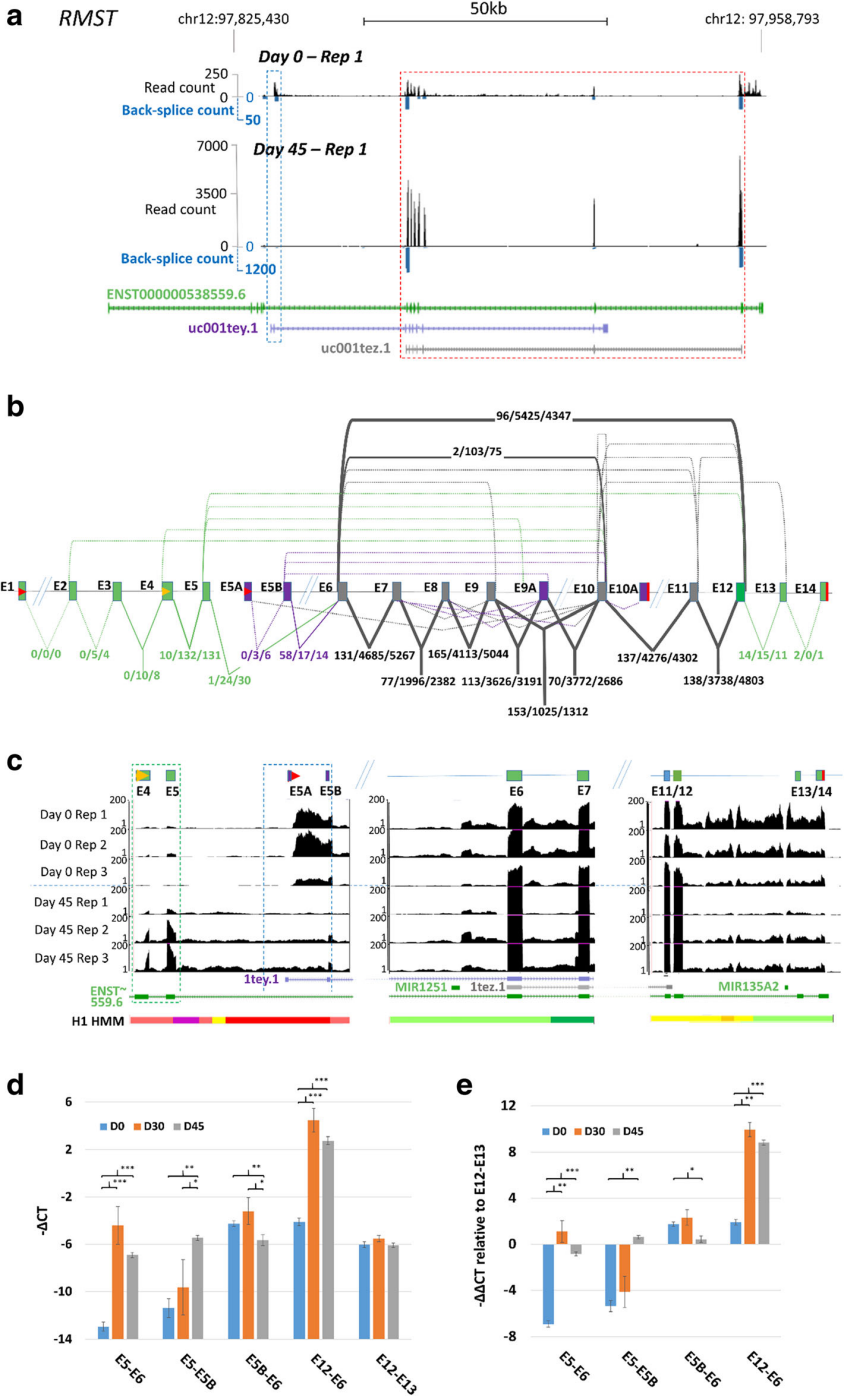
*RMST* is a circRNA in differentiated H9 cells. **a** Modified UCSC browser representation of mapped read count (black) and back-splice junction count (blue) at the *RMST* locus in untreated replicate 1 at day 0 and day 45. Back-splice reads span a single junction and only the donor and acceptor exons are indicated. Scales are different in the two time-points to allow visualisation of both. GENCODE (green) and Refseq (purple and grey) annotations are shown, together with genomic scale. Exons within circ*RMST*:E12-E6 are enclosed by a red box, upstream exons highly expressed at day 0 are enclosed by a blue box. **b** Schematic of *RMST* exon structure, showing circRNA junction counts (above exons) and canonical counts (below exons) summed across untreated replicates for all time-points (0/45/90). Counts were calculated for all exon combinations within each annotated transcript. Numbers are only shown for junctions with 10 or more supporting reads at 1 or more time-points. Exons specific to a single annotated transcript are colour coded (as in a). Exons present in multiple annotated transcripts are shown in grey. Exons are numbered relative to the longest ENCODE annotation ENST00000538559.6 (ENST~ 559.6). Annotated (red) and inferred (orange) transcription start and stop sites are also shown. Data from IGF-1 treated samples are comparable. **c** Adapted UCSC screen-shots showing changes in expression at exons of uc001tey.1 and ENST00000538559.6 upstream of E6 (left panel) and miRNA precursors flanking the E12-E6 circRNA (middle and right panels) in all untreated replicates. ENCODE/Broad chromatin state segmentation [52] within the H1 ES cell line is also shown (H1): Light red/bright red/purple = weak/active/poised promoter, light/dark green = weak/elongation transcription, orange/yellow = strong/weak enhancer). Scale is capped at 150 to allow direct comparison between time-points. **d**-**e** qPCR confirmation of increase in *RMST* circRNA and E5-E5B junctions. d. Mean ΔCt values from 3 biological replicates assayed at day 0, 30 and 45 are shown (+/− S.E.M). **e** ΔΔCt values relative to E12-E13. p-values *p < 0.05, **p < 0.01, ***p < 0.001

*RMST* read and circRNA junction counts were consistent between replicates at each time-point, and representative data from day 0 and 45 are shown in Fig. 5a. All exons corresponding to annotated isoform uc001tez.1 (grey) increased > 30 fold in abundance between these time-points, but there is an equivalent increase in a back-splice junction which spans all exons in this transcript. This is consistent with a single abundant circRNA (circ*RMST*:E12-E6, red box Fig. 5a and see Additional file 3: Figure S7). Strikingly, when junction counts across all time-points were analysed (Fig. 5b), only junctions internal to E6 and E12 (so expected to be within the circRNA) exceeded 150 counts at any time point, and most exceeded 3000 at days 45 and 90. An additional 18 low abundance *RMST* circRNAs were also identified, collectively encompassing all but the terminal annotated exons (Fig. 5b). Furthermore, exon junctions which both involve the main circRNA splice acceptor (E6) and could potentially be within linear RNAs (E5-E6 and E5B-E6), were at frequencies < 0.5% of E12-E6 at later time-points (e.g. 24 and 17 v 5425 at day 45).

To identify potential sites of transcript initiation, exons 5′ to the main circRNA were investigated (Fig. 5c), and uncovered both a high frequency of intron retention between exons 1 and 2 of uc001tey1 (defined here as E5A and E5B, blue box), and a reduction in reads mapping to this region by day 45. Over the same period there was an increase in reads and splice-junctions involving E4 and E5 (green box), indicating a change in isoform structure over time. These exons lie within a regions defined by ENCODE/Broad chromatin state segmentation [52] as a weak promoter (light red) within the human H1 ES cell line (Fig. 5c). In contrast, transcription immediately upstream and downstream of the E6-E12 region, which span miRNA precursors and could potentially be involved in generation of the circRNA, showed no consistent change (Fig. 5c middle and right hand panels).

To confirm changes in circRNA levels, and investigate the putative change in isoform levels, we used qPCR to assay splice junctions at days 0 and 45, and at an intermediate time-point (day 30) from the same differentiation series. Overall, the correlation between RNAseq junction counts and qPCR data was 0.93 (Additional file 3: Figure S8) and results are shown in Fig. 5d. A ~ 100 fold induction of E5-E6 (7-8 Ct) was observed between day 0 and 30, together with a similar increase in the E5-E5B junction by day 45 (both *p* < 0.001), the latter establishing that isoforms ENST00000538559.6 and uc001tey1 are not independent. A modest drop in E5B-E6 expression was also observed (*p* < 0.01 by day 45). The E12-E6 circRNA junction, present at similar levels to E5B-E6 at day 0, also increased > 100 fold by day 30 (*p* > 0.001). However, there was little change in the downstream canonical E12-E13 junction. The induction of both the E5A containing isoform, and the E12-E6 circRNA, is clear when the data is displayed relative to E12-E13 (Fig. 5e). In silico analysis of RNAseq data from human fetal and embryonic eye tissue (Mellough et al. in preparation) also established that the E12-E6 circRNA is the most abundant *RMST* isoform during differentiation in vivo (Additional file 3: Figure S9).

### The lncRNA *FIRRE* is downregulated, and upon differentiation its major transcripts are circular

**Fig. 6:**
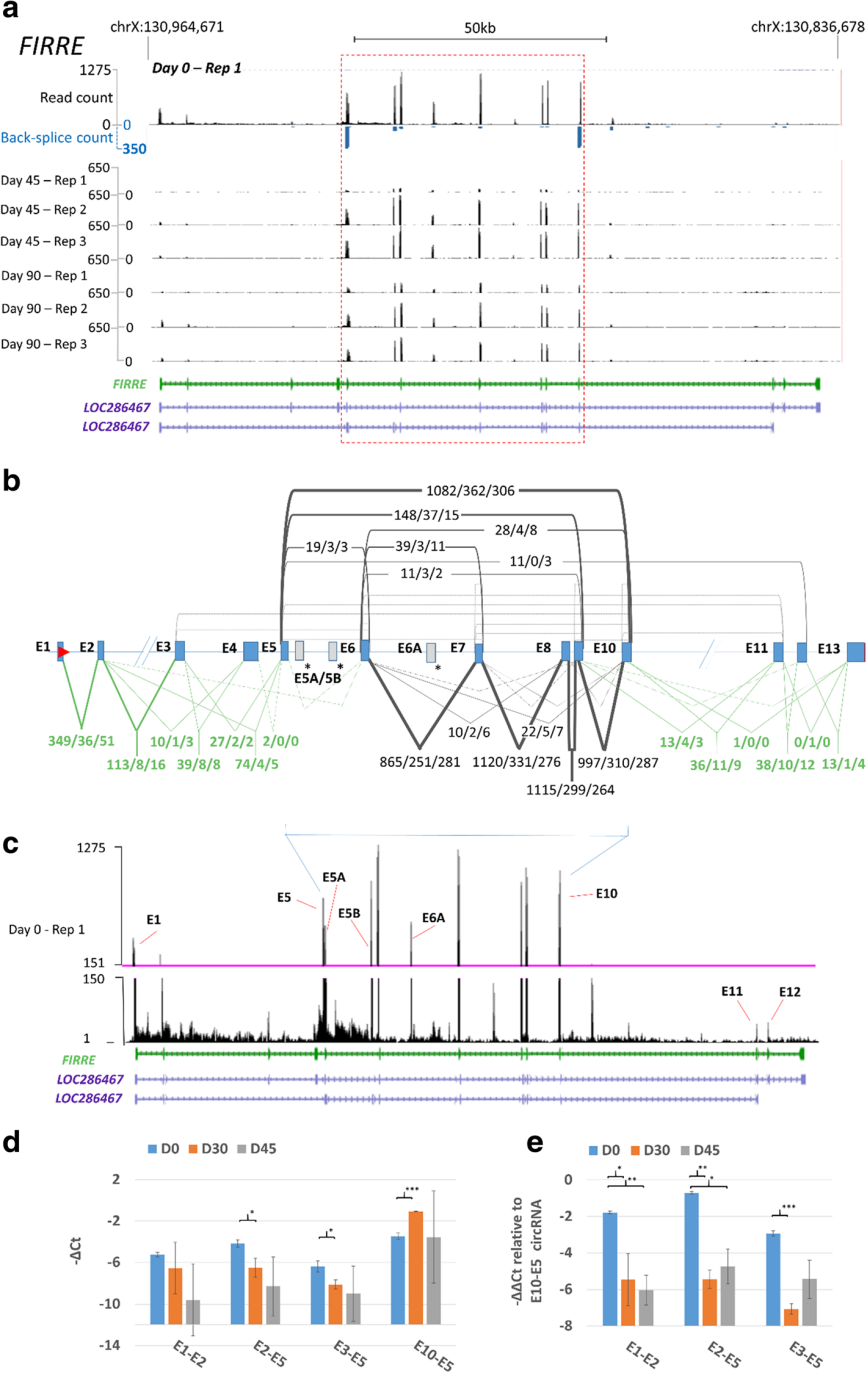
*FIRRE* is downregulated, and its major transcripts are circular in differentiated cells. **a** Modified UCSC browser representation of mapped read count coverage (black) and back-splice junction count (blue) at the *FIRRE* locus in untreated replicate 1 at day 0 (top), shown above the mapped read data from all untreated replicates at days 45 and 90. Back-splice reads span a single junction and only the donor and acceptor exons are indicated. GENCODE (green) and RefSeq (purple) annotations are also shown, together with a genomic scale. ENCODE/Broad chromatin state segmentation [52] within the H1 ES cell line is also shown: Light red/bright red/purple = weak/active/poised promoter, light/dark green = weak/elongation transcription, orange/yellow = strong/weak enhancer, grey = heterochromatin low signal, blue = insulator. **b** Schematic of *FIRRE* exon structure, showing circRNA junctions and counts (above exons) and canonical junctions and counts (below exons), summed across untreated replicates for all time-points (0/45/90). Additional exons not present within the GENCODE *FIRRE* annotation are shown in grey (asterisked) and numbered A, B etc. with respect to ENCODE annotation. Only junctions involving annotated *FIRRE* exons are shown, and numbers are only given for junctions with 10 or more supporting reads in 1 or more time-points. **c** Modified UCSC browser image showing position and relative abundance of additional exons not in ENCODE annotation. The scale is split at 150 to facilitate visualisation of rare exons (see Additional file 3: Figure S4). **d**-**e** qPCR validation of expression changes of circ*FIRRE*:E10-E5 and upstream junctions. d. Mean ΔCt values from 3 biological replicates assayed at day 0, 30 and 45 are shown (+/− S.E.M). e. ΔΔCt values relative to to E10-E5. *p < 0.05, **p < 0.01

*FIRRE* expression at day 0 was consistent between replicates, and exons with the highest RNAseq read counts (E5 to E10) were also bound by an abundant back-splice junction (Fig. 6a and see Additional file 3: Figure S7), indicative of a single dominant circRNA. Although expression fell in all replicates between day 0 and day 45, heterogeneity was observed, with replicate 1 showing a pronounced drop in expression of all exons (Fig. 6a). This is likely to reflect differences in activity of the single promoter region inferred from ENCODE data (Fig. 6a). In total, 20 *FIRRE* circRNAs were identified, ranging in abundance over 3 orders of magnitude (Fig. 6b) and encompassing all but 3 annotated exons (E1, E2, E13). All exon junction counts (Fig. 6b) were consistent with the general reduction in *FIRRE* expression between day 0 and 45. Critically, junction counts involving exons external to circRNAs (E1 and E2) fell more dramatically than others: Only the E10-E5 circRNA junction, and canonical junctions internal to these exons, exceeded 150 counts at days 45 and 90 (Fig. 6b). By day 45 the E10-E5 junction count (362) was ~ 10 fold higher than the E1-E2 junction count (32), and > 50 fold higher than any counts from junctions which could both involve the main circRNA acceptor exon (E5) and be within linear RNAs (E2-E5, 4; E3-E5, 2; E4-E5, 0). This suggests that in differentiating H9 ES cells, > 98% of reads from the abundant central exons are from circular RNAs.

The read distributions also suggested that *FIRRE* transcripts may contain additional exons which are either unannotated, or annotated within alternative transcripts (grey in Fig. 6b). We investigated these using a combination of annotation-free exon junction mapping and amplicon sequencing of RT-PCR products (see Methods). This both confirmed the existence of the rearranged transcript structures inferred from RNAseq data, and established that exons E5A, E5B, E6A, and the minor exon E10A and E10C, are integrated within multiple circRNAs up to > 1 kb in length (Additional file 3: Figure S10 and Additional file 6). Of the annotated *FIRRE* exons, only E1, E2, E4 and E13 were not identified within circRNAs by amplicon sequencing. This analysis also ruled out the existence of *FIRRE* transcripts containing upstream linear exons (e.g. E1, E2) spliced directly to unannotated exons downstream of E5 (Additional file 7), and identified a further 19 circRNA junctions and minor unannotated exons distal of E10 (Additional file 3: Figure S10). Interestingly, a dot-matrix of internal sequence repetition within *FIRRE* (Additional file 3: Figure S11) also established that the E5-E10 region contains the majority of the DCC repeats known to function as nuclear localisation signals [50, 53].

We used qPCR to validate the expression levels of E10-E5 and upstream exon junctions within RNAs isolated at days 0 and 45, together with an intermediate time-point, day 30 (Fig. 6). The correlation between RNAseq-derived junction counts and qPCR data across all *FIRRE* assays was 0.75, lower than for *RMST* (Additional file 3: Figure S8). This is likely due to the impact of repetitive sequence ([53] and Additional file 3: Figure S11) on both read mapping and PCR amplification. Despite this, the relative expression of all junctions at day 0 were consistent with the RNAseq data (Fig. 6d). We did observe an increase in E10-E5 expression by day 30, a time-point not assayed by sequencing, but all other junctions decreased in abundance. The relatively low expression level of all *FIRRE* exons in replicate A by day 45 (Fig. 6a) resulted in large standard errors of expression estimates at this time-point. However, relative expression levels of junctions remained consistent within replicates at each time-point, and the fall in E1-E2 and E2-E3 relative to E10-E5 observed in the RNAseq data was both confirmed, and found to be significant (~ 16-32X reduction, Fig. 6e). In silico analysis of RNA seq data from human fetal and embryonic eye tissue also established that the E10-E5 circRNA is the most abundant *FIRRE* isoform during differentiation in vivo (Additional file 3: Figure S9).

### *RMST* E12-E6 accounts for > 99% of adult neural *RMST* expression

**Fig. 7:**
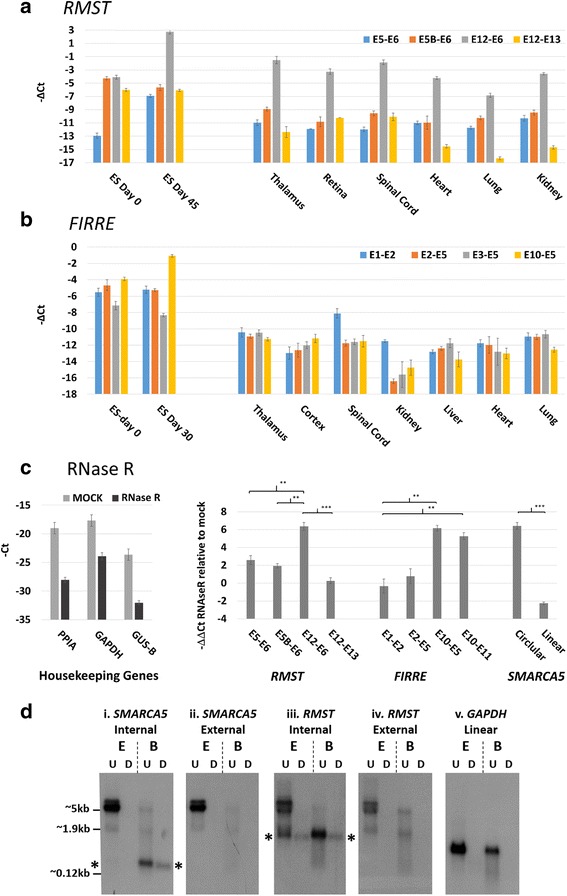
Tissue distribution and RNase R resistance of *RMST* and *FIRRE* junctions. **a** *RMST* junctions in adult tissue. **b** *FIRRE* junctions in adult tissue. Mean ΔCt values of 3 technical replicates are shown (+/− S.E.M). **c** RNase R sensitivity of *RMST* and *FIRRE* junctions. Left panel; –Ct values of 3 housekeeping genes in digested and mock reactions. Right panel; -ΔΔCt values of RNase R digested relative to undigested (see methods). A control linear/circRNA transcript pair (*SMARCA5*) is also shown. Means from 3 biological replicates are shown +/− S.E.M, except for *FIRRE* E2-E5 where only technical replicates were available. *p < 0.05, **p < 0.01, ***p < 0.001. **d** Replicate Northern blots of day 0 ES (E) and adult brain (B) RNAs, digested with RNase R (D) or undigested (U) and hybridised with i. *SMARCA5* probe internal to circRNA (E15-E16), ii. *SMARCA5* probe external to circRNA (E10-E13), iii. *RMST* probe internal to main circRNA (E6-E9), iv. *RMST* probe external to main circRNA (retained intron 5A-E5B), v. *GAPDH* probe (E3-E6). RNase R resistant transcripts are highlighted with asterisks, and approximate position of human rRNAs are shown

Having established that two functionally important lncRNAs almost exclusively generate circular isoforms during ES differentiation, we investigated their abundance in adult tissues (Fig. 7). In all neural tissues analysed as well as heart, the *RMST* E12-E6 circRNA junction was detected 7-8 cycles earlier than either of the linked upstream canonical junctions assayed (E5-E6, E5B-E6), indicating that it is also > 100 fold more abundant in these tissues (Fig. 7a). Read and back-splice distributions within independent publicly available total RNAseq data from adult brain [54] were also consistent with this (Additional file 3: Figure S12). In tissues such as lung and kidney the circRNA was ~ 15-50X more abundant, while in liver the linear transcripts could not be detected. In contrast, all *FIRRE* structures are present at much lower levels in the adult tissues analysed relative to ES cells (Fig. 7b), consistent with previous analyses [50]. Although all junctions were detected at low levels, E1-E2 was ~ 10X more abundant than other junctions in spinal cord, and the E10-E5 junction was very low in all tissues, indicating no circRNA enrichment. In silico analysis of independent publicly available RNAseq datasets also confirmed the low expression of all *FIRRE* exons in the adult tissues analysed here, as well as skin, breast, muscle, bladder, prostate, ovary, and colon ([54], data not shown).

### *RMST* and *FIRRE* exons external to circRNAs are sensitive to RNase R

To confirm that transcripts from both genes are circular in ES cells, we tested the prediction that circRNA specific exon junctions would be resistant to RNase R, using independent day 0 RNA isolations from the H9 cell line (Fig. 7c). As anticipated, RNase R digestion reduced levels of linear housekeeping genes ~ 100-500 fold (~ 7-9 cycles, left panel). In contrast, *RMST* E12-E6 and *FIRRE* E10-E5, together with *FIRRE* E10-E11 (which is present within 8 circRNAs), were enriched ~ 30-60 fold relative to linear housekeeping controls, significantly more than other junctions (*p* < 0.01 all comparisons). This is similar to the enrichment seen with a control circRNA (*SMARCA5* E16-E15 [7, 15]). However, the terminal exons assayed, which are not associated with any circRNAs (*RMST* E12-E13 and *FIRRE* E1-E2), showed no enrichment, confirming their linearity. Other proximal exons assayed either showed limited enrichment (2-4 fold *RMST* E5-E6, E5B-E6), or no enrichment (*FIRRE* E2-E5), suggesting they are also primarily within linear RNAs.

Finally, RNase R resistance of *RMST* E12-E6 was also confirmed by Northern blotting of RNA from day 0 ES cells and adult brain (Fig. 7d). A control probe internal to the circRNA from *SMARCA5* (E16-E15) confirmed effective RNase R digestion, as it identified a small RNase R resistant transcript abundant only in brain (Fig. 7d-i) which was not identified by a *SMARCA5* probe external to the circRNA (Fig. 7d-ii). This is consistent with RNAseq data (Additional file 3: Figure S12) and qPCR data (above). The *RMST* probe internal to the main E12-E6 circRNA (Fig. 7d-iii) identified a single RNase R resistant transcript as the dominant isoform in adult brain, also consistent with RNAseq data (Additional file 3: Figure S12) and qPCR data (above). Furthermore, this transcript was less abundant than other, longer, *RMST* transcript in ES cells, and was not identified by the *RMST* probe external to the E12-E6 circRNA (Fig. 7d-iv). The intensity of hybridisation to this transcript indicated some loss of material during RNase R digestion. However, loss was also observed with the *SMARCA5* circRNA control. These results provide additional evidence that *RMST* E12-E6 circRNA is the dominant isoform in adult brain. They also establish the presence of linear *RMST* isoforms over 5 kb in length within H9 ES cells, much longer than current annotations for this gene (~ 1.0-2.6 kb).

## Discussion

We have performed deep sequencing of a human ES cell retinal differentiation series to investigate changes in circRNA expression in a defined cell lineage, and to identify those of potential functional importance. We observed a striking transcriptome-wide increase in circRNA abundance specifically within the first 45 days, consistent with circRNA levels being under global control during differentiation. These results have parallels with temporal increases observed in previous analyses of tissues from a variety of species [32, 34, 37, 55], but the observation here of rapid circRNA increase upon differentiation, followed by relative stasis, is novel.

The most abundant circRNA we observed, and the one which showed the most significant change in expression over time, was from the lncRNA *RMST*, previously implicated in neuronal differentiation [48]. Surprisingly, we establish that > 99% of the transcriptional output from *RMST* in differentiating human ES cells, and a variety of adult tissues, is accounted for by this circRNA. This provides a simple explanation for the very weak signals obtained in Northern blots of PolyA+ RNA from brain during the original characterisation of this gene [56]. The *RMST*:E12-E6 structure was first described as an ES cell-specific trans-spliced transcript (*tsRMST*) and reported to play a role in maintenance of pluripotency [40]. However, it has since been shown to be both circular and abundant in differentiating and adult tissues ([11, 37], this study). It was identified as one of several hundred circRNAs expressed at > 50% of the level of associated linear isoforms in embryonic neuronal tissues [37]. In addition, its murine orthologue is abundant in mouse brain, RNase R resistant, and enriched in synaptoneurosomes [11]. Critically, the expression differential established here between circ*RMST*:E12-E6 and flanking exons suggests that the *RMST* isoform in differentiated cells warrants classification as a circular lncRNA, and that linear transcripts could represent by-products or unprocessed precursors. Our results also suggest that the circular isoform present in differentiating cells and adult tissues, and linear isoforms present in ES cells, are driven by distinct promoters.

We have also established that *FIRRE* is predominantly a circular lncRNA during human ES cell differentiation when it is expressed at high levels, and that the major circular isoform can account for > 98% of *FIRRE* transcription. This provides a simple explanation for the reported stability of mature *FIRRE* transcripts relative to pre-mRNAs [50], and for the larger changes in *FIRRE* expression observed in total RNA relative to oligodT enriched templates [51]. We have identified a total of 39 *FIRRE* circRNAs whose abundance spans 3 orders of magnitude, and confirmed the integration of novel unannotated *FIRRE* exons into circular transcripts. While the complexity of this locus has been noted [50], it is clear from our in silico analysis and amplicon sequencing that the structure and transcriptional output of this gene remains to be fully resolved. Furthermore, for both genes, sequence motifs and RNA binding proteins involved in circularisation, and the factors which control promoter activity and isoform changes, remain to be defined.

The huge differential in abundance of circular and linear exons we observed in differentiated time-points (average of >100X for *RMST* and > 30X for *FIRRE*) is presumed to be due to stability of circular forms and degradation of back-splice by-products. On an evolutionary timescale circularisation may provide a route through which the level of a lncRNA can be dramatically increased, due to the reduced degradation of circular forms, without altering promoter activity. It follows that subtle changes in the activity of promoters driving both genes could have profound effects upon circRNA levels and may, for instance, underpin the expression heterogeneity observed here for *FIRRE* at day 45. Our data also suggest that high sequencing depths will be required to accurately define the linear exons and transcription start sites of circular lncRNAs such as these and, together with the recent discovery that the archetypal circRNA ciRS-7 (*CDR1-AS*) is embedded within cryptic exons of a linear lncRNA [57], highlight the growing need for the integration of circRNA data within genome-wide annotations.

A key question to address is whether circularity merely serves to influence transcript abundance, or whether it can impact qualitatively upon function. While in vitro over-expression of both *RMST* and *FIRRE* have been shown to rescue some effects of loss or absence of endogenous gene expression [40, 48], the linear transcripts used in these analyses contained exons shown here to be almost exclusively circular at some time-points. As circularisation will alter sequence proximity and relative orientation, it could influence protein binding or binding partner position within RNA/protein complexes. Isoform differences could, therefore, account for the distinct roles in pluripotency [40] and neuronal differentiation [48, 58] attributed to *RMST*. Furthermore, circularity may increase complex stability, something particularly relevant to *FIRRE* which is involved in establishing and maintaining physical interactions between remote chromatin domains [59].

Our observation that circRNAs from non-coding RNAs account for a higher proportion of transcriptional output from their loci of origin than other circRNAs is also of interest, as the novel circular antisense RNAs we have identified may represent further circular lncRNAs of functional importance. However, non-coding RNAs are enriched for interspersed repeats which can promote circularisation [5, 8, 9], so it is possible that differences in the local genomic environment will contribute to these elevated circRNA levels.

## Conclusions

Finally, although there are a growing number of reports of functional circRNAs derived from coding genes [22, 60–62], some of the clearest evidence for circRNA function involves transcripts from non-coding loci. These include *CDR1-AS* and *CircPVT1*, which act as sponges for miR-7 and let-7 respectively, and have been implicated in zebrafish midbrain development [20] and cellular senescence [23]. In addition, *CircANRIL* has been shown to modulate rRNA maturation through binding to the 60S rRNA assembly factor *PES1* [29]. Current evidence suggests that the two lncRNAs defined here as being circular, function through distinct mechanisms: *RMST* is a transcriptional co-regulator which can bind to SOX2, hnRNPS2/B1, NANOG, and the PRC2 complex [40, 48], while *FIRRE* has been implicated in organising chromosomal domain topology, and tethering the inactive X chromosome to the periphery of the nucleolus through interaction with CTCF [50, 51]. Our results therefore also extend the range of molecular mechanisms through which circRNAs are known to function.

## Methods

### Source of human materials used

All tissue RNAs, and the control human DNA, were obtained as purified nucleic acids from Biochain (AMS Biotechnology).

### Differentiation of H9 ESCs

Passage 34-35 of the human embryonic stem cell line H9 line (obtained from WiCell, agreement number 06-W097), were expanded and differentiated according to the protocol outlined in [41], with minor modifications [42] to generate 3D laminated retina containing the major retinal cell types including photoreceptors. Cells were differentiated in triplicate with and without insulin growth factor 1 (IGF-1) treatment [42]. Upon differentiation, hESCs formed embryoid bodies (EBs) which developed morphologically distinct phase-bright structures reminiscent of the evaginating optic vesicle and invaginating optic cup, as previously described [42]. Phase-bright structures were first observed to develop around the periphery of differentiating EBs, with optic vesicles arising as early as day 15 and optic cups developing by day 45. The retinal identity of these structures was determined by immunocytochemistry: Retinal organoids were cryosectioned and reacted against Pax6 (Covance PRB-278P, 1:200), HuC/D (Invitrogen A21271, 1:100), Crx (Abnova H00001406-M02, 1:200) and Recoverin (Millipore AB5585, 1:1000) antibodies as previously described [42]. Images were obtained using a Zeiss Axio Imager.Z1 microscope with ApoTome.2 accessory equipment and AxioVision or Zen software.

### High-throughput RNA sequencing

Cells were harvested at multiple time points without any physical selection for embryoid bodies, to ensure cells harvested were representative of the whole population. RNA was extracted using the RNAeasy micro extraction kit with DNA elimination columns according to manufacturer’s recommendations. RNA was quantified using the NanoDrop ND-1000 spectrophotometer (Thermo Scientific), and RNA quality was assessed using the Agilent 2100 Bioanalyser (Agilent Technologies). RIN values indicated some reduction in RNA quality over time, with day 0 sample RIN values ranging from 9.8-10, day 45 ranging from 7.6-9.1 and day 90 ranging from 7.1-8.9. RNAs were ribosome depleted and sequenced by AROS Applied Biotechnology (Aarhus, Denmark), using the TruSeq RiboZero Stranded Total RNA LT kit (Illumina) to generate paired-end 100 bp sequence libraries as described previously [7].

### Sequence references

Genome and transcriptome FASTA files for human (HG19) were obtained from the UCSC genome browser [63]. Aligner-specific index files were built for STAR (command: STAR —runThreadN 8 —runMode genomeGenerate —genomeFastaFiles $hg19_dir —genomeDir $output_dir), and Bowtie1 (command: bowtie-build hg19.fa hg19) and Bowtie2 (bowtie2-build hg19.fa hg19) aligners.

### Mapping

The quality of sequenced reads was checked with FASTQC (http://www.bioinformatics.babraham.ac.uk/projects/fastqc/). Sequence reads were first mapped to the genome using Bowtie2 [64] to derive inner distance metrics prior to Tophat [65] runs. Metrics were calculated using *CollectInsertSizeMetrics.jar* from Picard (https://sourceforge.net/projects/picard/). Parameters for Tophat runs were: —library-type fr-firststrand, —no-coverage-search, —b2-sensitive, —microexon-search and -× 20. For alignments to the genome using STAR [66] parameters used were: —outFilterMultimapNmax 7 and —outFilterMismatchNmax 2. Annotation free canonical splice-junction mapping was performed, where indicated, with MapSplice 2.0 [67] using default parameters.

### circRNA (back-splice) and canonical splice detection

After modifying read ids, PTESFinder v.1 [46] was used to screen all reads from each sample for back-splice exon junctions within the GENCODE v19 human reference transcript set, using the following parameters: JSpan = 8, PID = 0.85, segment size = 60 and m = 7. Analyses were guided by supplying FASTA sequences of previously identified back-splice junctions (*n* = 40,594). PTESFinder was also used for annotation-free identification of back-splice junctions against the HG19 human reference genome sequence (modified scripts available on request).

### Analysis of RNA editing sites

RNA editing sites were identified in 1000 bp regions flanking circRNA back-splice junctions by cross-referencing genomic coordinates of published RNA editing sites (rnaedit.com [68, 69]) using intersectBed and closestBed from BEDTools. Data from circRNA generating genes identified only in day 0 only were then compared to circRNA producing genes identified only in differentiated cells (day 45 and 90).

### Gene expression estimates

HTSeq [70] and Cufflinks [71] were used to quantify transcripts. Library size normalisation and statistical tests of differential expression (based on raw counts produced using HTSeq) were performed using the DESeq2 [72] package in R (https://www.r-project.org). Splice site expression values were calculated as Junctions per Million (JPM) by dividing each junction count by total counts per sample (canonical + back-splice) and multiplying by 10e6. Gene ontology analyses were performed using the Panther classification system [73].

### Visualisation

BigWig files were generated from alignments to the genome using *genomeCoverageBed* from BEDtools and *bedGraphToBigWig* from UCSC, and visualised using Galaxy, a web-based tool for sequence analysis [74]. Distributions of aligned reads were visually examined on the integrative genomics viewer [IGV v 2.1.21 [75]] and on the UCSC genome browser. Dot matrix analyses were performed using YASS [76] with default parameters. Exon junction heat maps were generated using junction counts normalised to total read counts per sample, and mean expression value of each transcript across samples.

### Statistical analysis of back-splice abundance

To identity significant changes in circRNA abundance, two tests for differential expression (DE) were performed (see Additional file 3: Figure S5):

#### Sample level DE analysis

To account for differences in library size and transcriptome-wide changes in circRNA expression levels between pairs of time points, contingency tables for each circRNA were constructed consisting of circRNA junction counts in both time points, versus total junction counts (circular and canonical) minus the circRNA junction counts for the circRNA being tested.

#### Locus level DE analysis

To control for locus specific changes in total gene expression between time points, contingency tables for each circRNA were constructed using only junction counts from the locus of origin. These consisted of circRNA junction counts in time points A and B, versus canonical junction counts from all annotated exons from the locus being tested.

For both analyses, Fisher’s exact tests were first performed for each circRNA using contingency tables generated from summed read counts across replicates. To control for heterogeneity, t-tests were then performed on all the significant circRNAs, using read count data from each replicate normalised against total sample read count (Sample-level analysis) and against total read count from each transcript (locus level analysis). A Benjamini-Hochberg false discovery rate of 0.05 was used as threshold throughout. Expression heat maps were generated from circRNA junction counts within untreated samples following removal of transcripts present in segmental duplications. Read counts were subject to sample normalisation relative to total read count, and transcript normalisation relative to mean junction count across samples.

### Quantitative PCR (qPCR)

cDNA was synthesized using high-capacity cDNA kits (Applied Biosystem) with random hexamers according to manufacturers recommended protocols. Quantitative PCR experiments were performed using Taqman master mix (Life Technologies). Primers and probe sequences are given in Additional file 8. Transcript expression was normalized using the ∆Ct method relative to the geometric mean of 3 housekeeping genes (*GAPDH*, *PPIA* and *GUSB*) analyzed using TaqMan gene expression assays (Applied Biosystems) as described previously [7]. Significance of expression differences was assessed using 2 tailed t-tests.

### RNase R digestion

For qPCR, one microgram of RNA was added to 1 μl of 10× RNase R buffer and 20 units of RNase R (Epicentre), or zero units for mock treatment, in a 10 μl reaction volume. Tubes were then incubated at 37 °C for 30 min. For Northerns, one unit of enzyme was used per μg of RNA, and reactions were stopped by incubation at 70 °C for 15 min.

### Northern analysis

Replicate sample sets consisting of 6 μg of RNAse R digested and undigested total RNAs from Day 0 H9 ES cells and adult brain were loaded on to a 0.9% formaldehyde gel, electrophoresed, and blotted onto a Hybond N nylon membrane (GE Healthcare). Probes were generated using exon specific primers (see Additional file 8) to amplify day 0 cDNA and purified by 2 X 30 cycles of PCR. 50 ng of probe was labelled by random priming with α- 32-P dNTPs (Megaprime DNA labelling system, GEHealthcare) and hybridized overnight at 65 °C in Church and Gilbert solution. Membranes were washed for 10 min at room temperature in 2XSSC (3X) followed by 10 min at 65 °C in 1XSSC/0.1% SDS (2X), and 45 min at 65 °C in 0.1XSSC, 0.5% SDS. Membranes were exposed to X-ray film with an intensifying screen at − 70 °C.

### Amplicon sequencing of *FIRRE* transcripts

Exon-specific primers linked to Illumina sequencing adapters were designed to generate amplicons spanning potentially novel exons using cDNA from day 0 (see Additional file 6). Primers were used in both convergent and divergent orientation to enable amplification of both circular and linear isoforms. Illumina sequencing indexes were added to each amplicon using a Nextera indexing kit (Illumina), and amplicons were sequenced to a target depth of 1500-6000 reads (depending on the number and intensity of visible products) using a MiSeq Nano Kit (Illumina) all according to manufacturer’s recommendations.

## Additional files

Additional file 1: Sequencing Quality Control. Data shows Illumina quality control metrics. PF = Passing chastity filter. Q30 = Phred quality score of 30 or above. (XLSX 9 kb)

Additional file 2: a-d. Gene Ontology analyses showing over and under-represented gene ontologies (biological process and molecular function) in genes differentially expressed between untreated time-points. Analyses were performed using the Panther classification system [73], with a Bonferroni correction for multiple testing. e. Genes differentially expressed between IGF-1 treated and untreated samples. List includes genes identified by comparision at each timepoint, and an all v all comparison. No significant genes were identified in the day 90 analysis. (XLSX 50 kb)

Additional file 3: **Figure S1.** Expression of genes implicated in pluripotency, eye field formation, and circRNA biogenesis. **Figure S2.** Intersection of structures identified by PTESFinder relative to other commonly used circRNA identification methods. **Figure S3.** Heat map showing genes differentially expressed between time-points and treatment. **Figure S4.** ES cell specific circRNAs are low frequency structures derived from highly expressed genes. **Figure S5.** Flow chart showing statistical analysis of back-splice abundance. **Figure S6**. Relative expression of canonical and back-splice junctions in DE genes. **Figure S7.** Identification of abundant *RMST* and *FIRRE* back-splice junctions using other in silico methods. **Figure S8.** Correlation between qPCR and RNAseq data. **Figure S9.** Junction counts of major *RMST* and *FIRRE* circRNAs in human embryonic / fetal samples. **Figure S10**. Additional circRNAs defined by exons not present in *FIRRE* annotation and confirmation of exon integration. **Figure S11.** The E10-E5 circRNA contains *FIRRE* DDC repeats. **Figure S12.** Confirmation of *RMST* isoform differences in independent adult datasets. (PDF 2416 kb)

Additional file 4: CircRNA transcripts showing significantly altered expression in both locus and sample level analyses. Normalised circRNA and canonical RNA junction read counts in all control sample replicates are shown, together with average abundance ratio (circRNA junction frequency/canonical junction frequency), genomic co-ordinates, and circbase i.d.s where available. The most significant *p*-values obtained in each group of pair-wise t-tests (sample DE and locus DE) are also shown for each transcript. Analyses in which transcript was identified are indicated as follows; U (untreated), I (IGF-1 treated), B (Both analyses). For details of analysis, see text and methods. (XLSX 115 kb)

Additional file 5: Gene Ontology analysis showing over and under-represented gene ontologies (biological process and molecular function) in parent loci of 239 significantly altered circRNAs. Analyses were performed using the Panther classification system [73], with a Bonferroni correction for multiple testing. (XLSX 11 kb)

Additional file 6: *FIRRE* exon junctions confirmed by amplicon sequencing. All reads were mapped against hg19 without reference to transcript annotation: Amplicons using convergent primer pairs and divergent primer pairs (to amplify circRNAs only) are shown separately. Canonical junctions were mapped using MapSplice [67], circRNA (back-splice) junctions were mapped using PTESFinder [46]. For details, see methods. Exon number is according to schema in Fig. 6b. Junction position (hg19), amplicons of origin, and junction frequencies, are given for all junctions. Only splices with a frequency of 1% or higher in each amplicon, identified either by MapSplice or PTESfinder, are reported. Canonical junctions present within the current *FIRRE* annotation are show in blue. All others are not present within current annotation. Off target junctions (presumed to be generated by illegitimate primer binding) are also shown. Data is for confirmation of junction presence within transcripts only: Junction frequency is affected by position relative to primer, size dependent amplification bias during Nextera indexing, and size dependent bias in cluster formation/resolution efficiencies during MiSeq sequencing. (XLSX 71 kb)

Additional file 7: Genomic co-ordinates of all annotated and novel *FIRRE* exons. The position of all junctions are shown with respect to hg19, together with their current annotations where appropriate. Splice acceptor and donor junctions identified by annotation-free mapping of day 0 samples against hg19 using MapSplice are shown, together with donor exons and acceptor exons, and junction numbers in brackets. Supporting evidence for each junction is colour coded. Minor / Others – additional junctions not involving annotated exons which are present at low frequency. (XLSX 12 kb)

Additional file 8: Sequence of all primers generated for this study, together with qPCR probes, amplification efficiencies, and primer/probe combinations used. Exon content of amplicons used for Northern analyses is also shown. For additional assays, see [7]. (XLSX 12 kb)

## Abbreviations

circRNA: Circular RNA
DE: Differential expression
ES cell: Embryonic stem cell
hnRNPs: Heterogeneous nuclear ribonuncleoproteins
IQR: Inter quartile range
lncRNA: Long non-coding RNA
