# Supplementary material for: Analysis of human ES cell differentiation establishes that the dominant isoforms of the lncRNAs RMST and FIRRE are circular
Source: BMC Genomics. 2018 Apr 20;19:276. doi: 10.1186/s12864-018-4660-7 (PMC5910558; doi:10.1186/s12864-018-4660-7)
Supplement: Supplementary file 3 — Figure S1. Expression of genes implicated in pluripotency, eye field formation, and circRNA biogenesis. Figure S2. Intersection of structures identified by PTESFinder relative to other commonly used circRNA identification methods. Figure S3. Heat map showing genes differentially expressed between time-points and treatment. Figure S4. ES cell specific circRNAs are low frequency structures derived from highly expressed genes. Figure S5. Flow chart showing statistical analysis of back-splice abundance. Figure S6. Relative expression of canonical and back-splice junctions in DE genes. Figure S7. Identification of abundant RMST and FIRRE back-splice junctions using other in silico methods. Figure S8. Correlation between qPCR and RNAseq data. Figure S9. Junction counts of major RMST and FIRRE circRNAs in human embryonic / fetal samples. Figure S10. Additional circRNAs defined by exons not present in FIRRE annotation and confirmation of exon integration. Figure S11. The E10-E5 circRNA contains FIRRE DDC repeats. Figure S12. Confirmation of RMST isoform differences in independent adult datasets. (PDF 2416 kb) [file 12864_2018_4660_MOESM3_ESM.pdf]

Supplementary Figures Table of Contents

Legends to Supplementary Figures S1-S12 **2-3**

Figure S1: Expression of genes implicated in pluripotency, eye field formation, and circRNA biogenesis **4**

Figure S2: Intersection of structures identified by PTESFinder relative to other commonly used circRNA identification methods **5**

Figure S3: Heat map showing genes differentially expressed between time-points and treatment **6**

Figure S4. ES cell specific circRNAs are low frequency structures derived from highly expressed genes **7**

Figure S5: Flow chart showing statistical analysis of back-splice abundance. **8**

Figure S6: Relative expression of canonical and back-splice junctions in DE genes **9**

Figure S7: Identification of abundant *RMST* and *FIRRE* back-splice junctions using other *in silico* methods **10**

Figure S8. Correlation between qPCR and RNAseq data **11**

Figure S9. Junction counts of major *RMST* and *FIRRE* circRNAs in human embryonic/ fetal samples **12**

Figure S10: Additional circRNAs defined by exons not present in *FIRRE* annotation and confirmation of exon integration **13**

Figure S11. The E10-E5 circRNA contains *FIRRE* DDC repeats **14**

Figure S12. Confirmation of *RMST* isoform differences in independent adult datasets **15**

Supplementary References **16**

Figure S1: Expression of genes implicated in pluripotency, eye field formation, and circRNA biogenesis. Expression levels in untreated samples are shown, together with p-values from t-tests comparing means in day 0 versus day45/90 (pluripotency and circRNA genes) and day0/45 v day 90 (eye field genes). **A.** *NANOG* (1), **B.** *MITF* (2), **C**. *GNAT1* (3), **D**. *BEST1* (4), **E**. *RCVRN* (5), **F** *IMPG1* (6), **G** *QKI* (7), **H** *MBLN1*, **I** *MBLN3*.

Figure S2: Intersection of structures identified by PTESFinder relative to other commonly used circRNA identification methods. Upset plot (8) of structures identified using PTESFinder [PF (9)], Find_Circ [FC (10)], CIRCexplorer [CE (11)] and CircRNA_Finder [CF (12)]. The largest intersecting set of structures were identified by all 4 methods. To enable like for like comparison, PTESFinder was adapted (v2) to perform unguided back-splice identification without transcript annotation.

Figure S3: Heat map showing genes differentially expressed between time-points and treatment. Transcripts with a total count of less than 100 reads across all samples (i.e. replicates, time-points and treatments) were excluded from the analysis. For details, see Methods.

Figure S4: ES cell specific circRNAs are low frequency structures derived from highly expressed genes. A. Read count frequency of circRNAs identified in **i)** ES cells only, **ii)** untreated differentiated cells only (days 45/90), **iii)** both ES and untreated differentiated cells. CircRNAs specific to day 0 were supported by an average of only 1.36 junction reads per circRNA (SD 1.34, median 1), compared to 19.5 (SD 86.5, median 5) for those identified in both day 0 and subsequent time-points, and the vast majority of structures with read counts >10 at any time point are identified in both ES and differentiated cells. Only 4 ES cell-specific structures with a total circRNA read count >10 were identified, and all were found at very low frequencies relative to canonical junctions (<1%) in genes/isoforms with ~100 fold higher expression in day 0 than in day 45; *DIAPH2* (2 structures), *USP44* and *POLGR3*. None are strong candidates for ES-specific functional circRNAs. **B. i) Example of ES specific structure.** Modified UCSC browser images of ES-specific circRNAs from *DIAPH2* showing read count frequencies at day 0 and day 45 untreated (replicate 1). All *DIAPH2* circRNAs involve exons present within an abundant short isoform, and circRNA junctions represent <1% of junction levels in this isoform. The isoform has the capacity to encode a protein containing the diaphanous autoregulatory domain (DAD) without the formin homology or Rho GTPase-binding domains. **ii)** The short isoform includes an un-annotated first exon (left panel) and an unannotated 3' splice site at the end of intron 1 (right panel). The abundance of this isoform falls >100X between day 0 and day 45.

Figure S5: Flow chart showing statistical analysis of back-splice abundance. Two tests were performed to control for tanscriptome-wide effects and locus specific changes is total expression. Each used Fisher's exact tests to identify putative differentially expressed genes, followed by T-tests to control for heterogeneity between replicates. For details, see Methods.

Figure S6: **Relative expression of canonical and back-splice junctions in DE genes.** Heat maps showing relative expression of canonical junctions (left panel) and back-splice junctions (right panel) from ~ 2000 transcripts identified as differentially expressed in both sample-level and locus-level t-tests, when comparing day 0 samples against day 45/day 90 untreated samples combined. For details, see text and methods.

Figure S7: Identification of abundant *RMST* and *FIRRE* back-splice junctions using other *in silico* methods. Junction counts for a. *RMST* E12-E6 and b. *FIRRE* E10-E5 are shown for all 4 circRNA identification tools: PTESFinder (9), Find_Circ (10), CIRCexplorer (11) and CircRNA_Finder (12). Error bars represent S.E.M.s of replicate data at each time-point.

Figure S8. Correlation between qPCR and RNAseq data. a. *RMST* **b.** *FIRRE*. Plots show DCt values for all qPCR assays plotted against Log_2 RNAseq-derived splice junction counts normalised for library size. Slopes and correlation coefficients are shown.

Figure S9. Junction counts of major *RMST* and *FIRRE* circRNAs in human embryonic / fetal samples. a. *RMST* E12-E6, **b.** *FIRRE* E10-E5. In both plots RNAseq derived circRNA junction counts from human eye/retinal tissue samples isolated prior to post coital week 12 (~84 days) are shown, together with the average counts of canonical junctions internal to each circle, and the average counts of canonical junctions external to each circle. All counts were normalised to library size. *RMST* E12-E6, and the canonical junctions internal to this circRNA, are ~50-200 times more abundant than exon junction external to the circRNA in all samples. *FIRRE* E10-E5 junctions and internal canonical junctions are ~10-50X more abundant than external junctions, and the expression of all *FIRRE* exons drops over time. These results are consistent with our ES cell data. However, the reduction in *FIRRE* expression over time is more pronounced in the primary tissue than in the ES model. For *FIRRE*, only contiguous exons not separated by major unannotated exons (E7-E10) were used to calculate average internal junction count. No *FIRRE* E10-E5 junctions were detected in the day 56 sample. All data are from GEO study GSE98370 (Mellough et al. Manuscript in preparation): All RNAseq samples were extracted from total eye preparations, with the exception of those at days 53, 63, 70 and 84 which were from retinal tissue.

Figure S10: **Additional circRNAs defined by exons not present in *FIRRE* annotation and confirmation of exon integration. A.** Schematic diagram of *FIRRE* exons including novel, low abundance, and 3' exons. Total circRNA junction counts identified by using PTESFinder to map reads from all untreated samples against hg19, without reference to annotation, are shown. Those with >10 supporting reads are shown above the exons, those with <10 below the exons. Exons in *FIRRE* annotation are shown in blue. **B**. Modified UCSC screen-shot showing read abundance (black) and CircRNA junctions (red) in 3' region of gene at Day 0 in replicate 1. Peak heights associated with all exons distal of E10, both annotated and inferred from PTESFinder/RT-PCR, are indicated. **C.** Amplicons generated from ES cell day 0 RNA (cDNA), genomic DNA (gDNA) and no template control (-ive) are shown, along with primers used and molecular weight markers. All primer combinations in both convergent and divergent (5BF+5R, 6F+5R) orientations generate cDNA specific products. Amplicons generated with MiSeq adapters (suffix _MS) were sequenced to confirm presence of specific exon junctions (see Additional File 5).

Figure S11. The E10-E5 circRNA contains *FIRRE* DDC repeats. Dot-matrix self-comparison (see methods) showing *FIRRE* transcript with additional abundant exons included (5A, 5B, 6A). Position of exons is shown, with the span of E10-E5 circRNA highlighted in red.

Figure S12. Confirmation of *RMST* isoform differences in independent adult datasets. **A.** Modified UCSC screen-shot showing read distribution across *RMST* in H9 total RNA at day 0 and day 45 untreated, compared to RNAseq data from H9 PolyA+ RNA [merged data from (13)], and both total RNA and polyA+ RNA from brain [merged data from (14)]. Only exons internal to circ*RMST*:E12-E6 are abundant in the total RNA sample from adult brain, but exons upstream and downstream of the circRNA are observed in polyA+ RNA from both adult brain and H9 cells at day 0. **B-C.** Read distributions in known circRNA generating genes *SMARCA5* and *MAN1A2*. Donor and acceptor exons of most abundant circRNA in each gene are indicated with red diamonds. Exon structure of Refseq or ENCODE transcripts are shown in each panel.

Figure S1

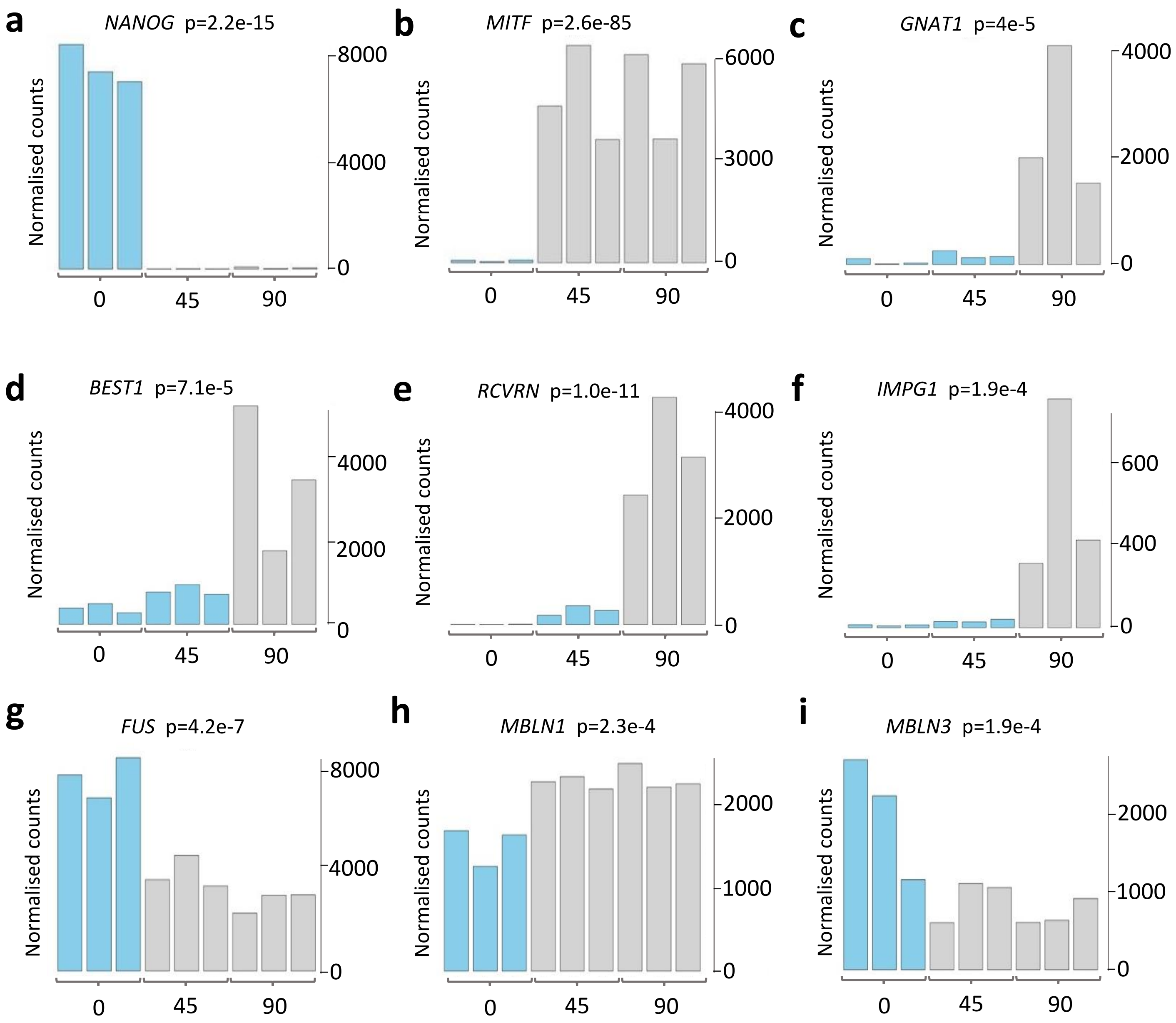
a
NANOG p=2.2e-15
Normalised counts
8000
4000
0
0
45
90
b
MITF p=2.6e-85
Normalised counts
6000
3000
0
0
45
90
c
GNAT1 p=4e-5
Normalised counts
4000
2000
0
0
45
90
d
BEST1 p=7.1e-5
Normalised counts
4000
2000
0
0
45
90
e
RCVRN p=1.0e-11
Normalised counts
4000
2000
0
0
45
90
f
IMPG1 p=1.9e-4
Normalised counts
600
400
0
0
45
90
g
FUS p=4.2e-7
Normalised counts
8000
4000
0
0
45
90
h
MBLN1 p=2.3e-4
Normalised counts
2000
1000
0
0
45
90
i
MBLN3 p=1.9e-4
Normalised counts
2000
1000
0
0
45
90

Figure S2

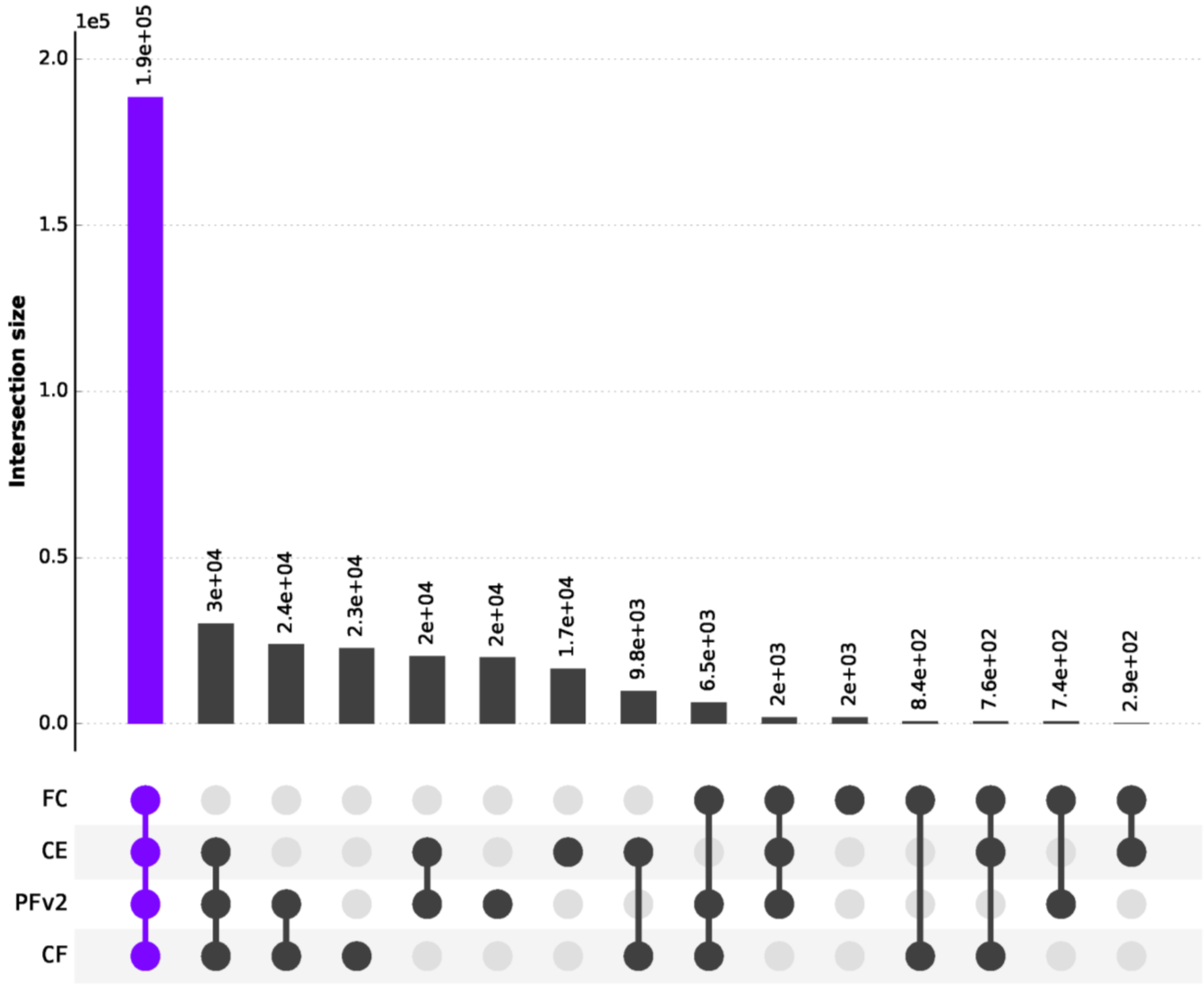
1e5
2.0
1.5
1.0
0.5
0.0
Intersection size
1.9e+05
3e+04
2.4e+04
2.3e+04
2e+04
2e+04
1.7e+04
9.8e+03
6.5e+03
2e+03
2e+03
8.4e+02
7.6e+02
7.4e+02
2.9e+02
FC
CE
PFv2
CF

Figure S3

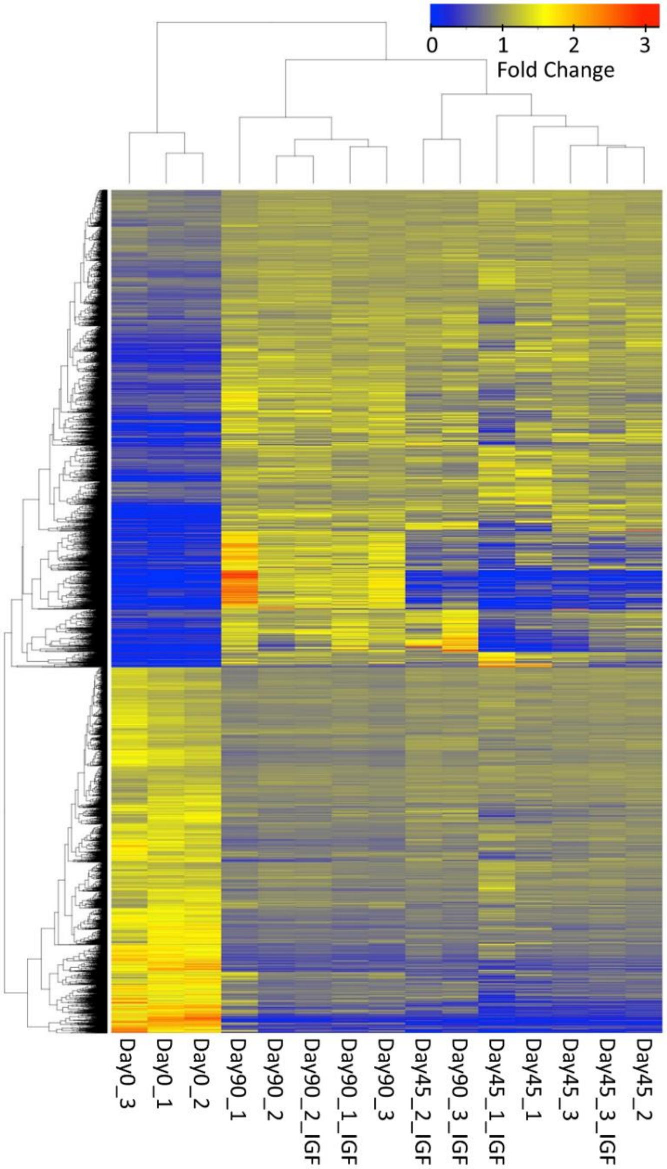

0
1
2
3
Fold Change
Day0_3
Day0_1
Day0_2
Day90_1
Day90_2
Day90_2_IGF
Day90_1_IGF
Day90_3
Day45_2_IGF
Day90_3_IGF
Day45_1_IGF
Day45_1
Day45_3
Day45_3_IGF
Day45_2

Figure S4

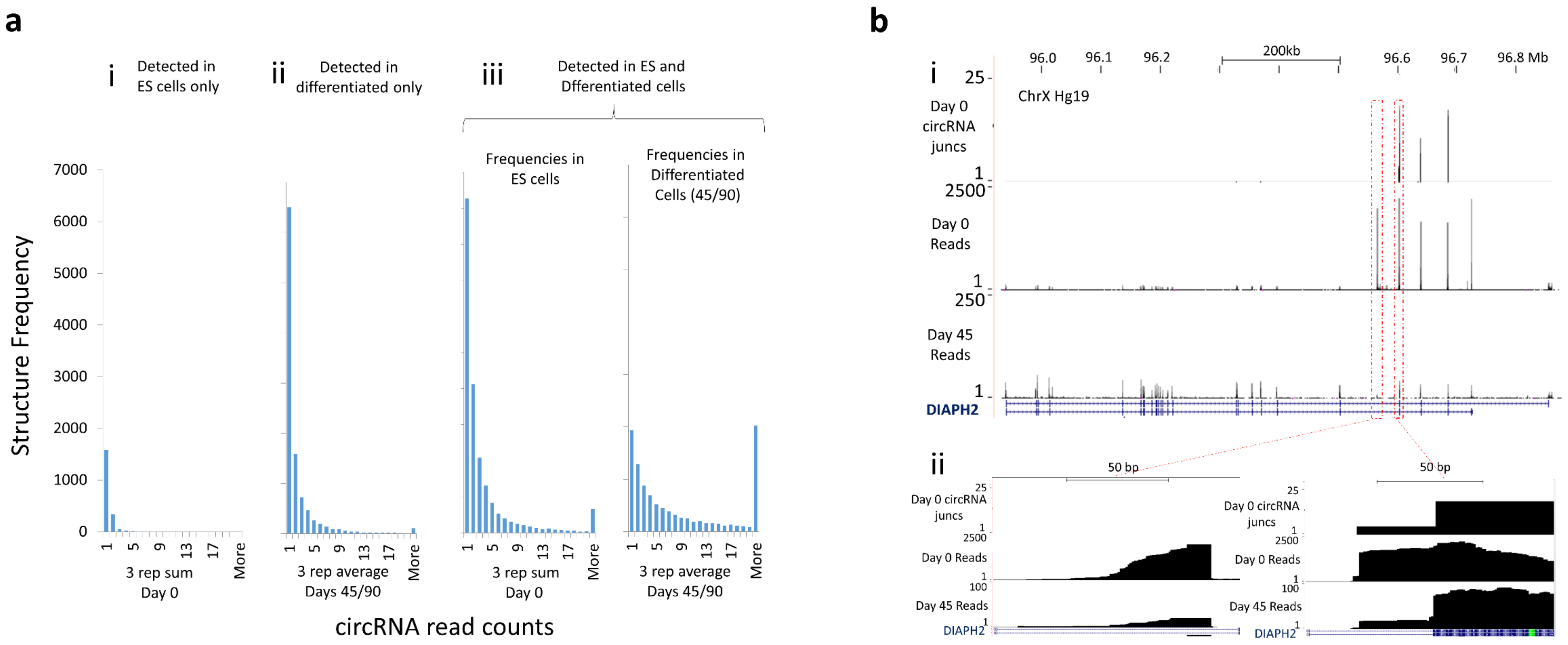
a
i Detected in ES cells only
ii Detected in differentiated only
iii Detected in ES and Dfferentiated cells
Frequencies in ES cells
Frequencies in Differentiated Cells (45/90)
Structure Frequency
7000
6000
5000
4000
3000
2000
1000
0
1
5
9
13
17
More
3 rep sum Day 0
3 rep average Days 45/90
3 rep sum Day 0
3 rep average Days 45/90
circRNA read counts
b
i
96.0
96.1
96.2
200kb
96.6
96.7
96.8 Mb
ChrX Hg19
Day 0 circRNA juncs
Day 0 Reads
Day 45 Reads
DIAPH2
25
1
2500
250
ii
50 bp
Day 0 circRNA juncs
Day 0 Reads
Day 45 Reads
100
DIAPH2

Figure S5

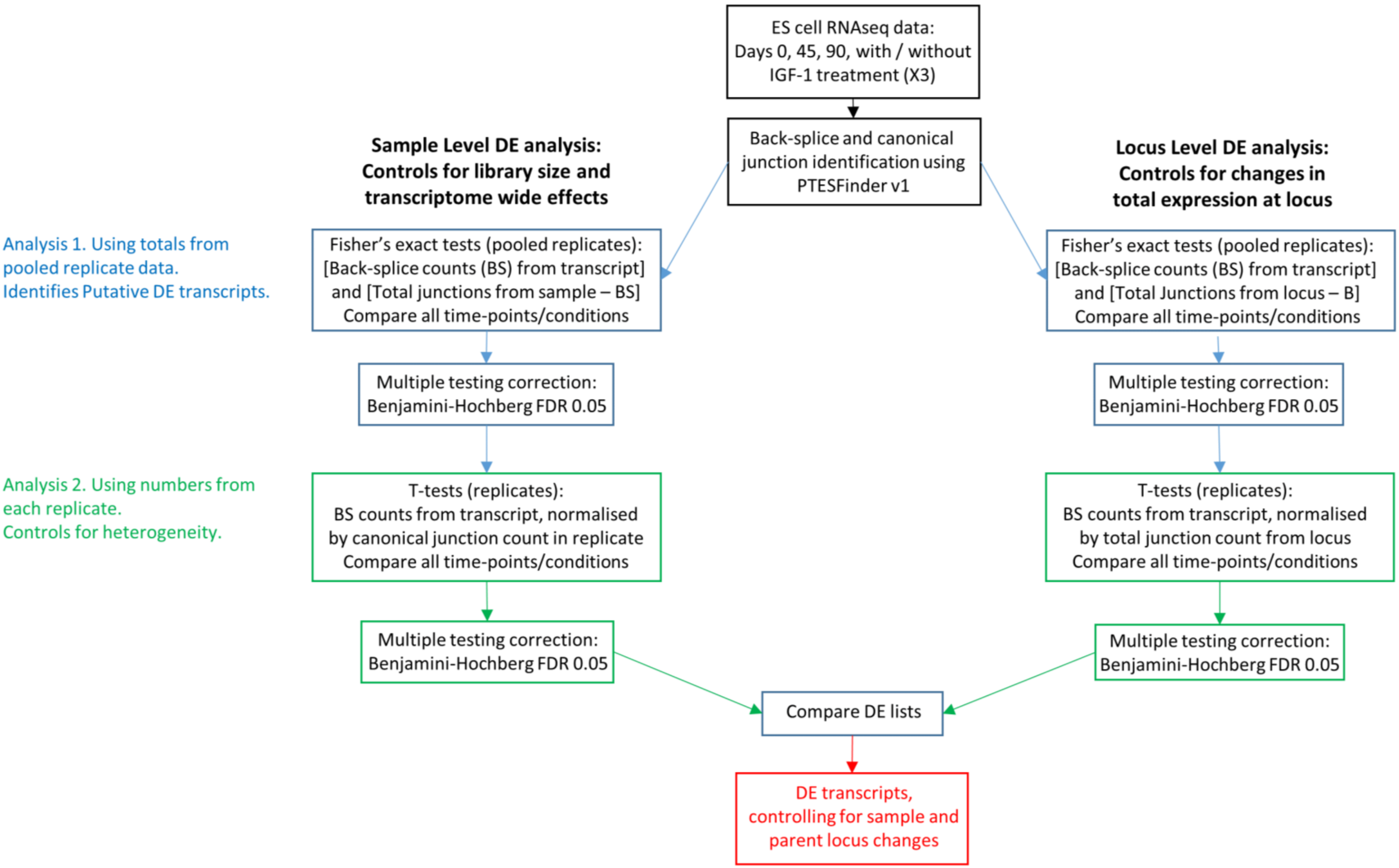
ES cell RNAseq data:
Days 0, 45, 90, with / without
IGF-1 treatment (X3)
Back-splice and canonical
junction identification using
PTESFinder v1
Sample Level DE analysis:
Controls for library size and
transcriptome wide effects
Locus Level DE analysis:
Controls for changes in
total expression at locus
Analysis 1. Using totals from
pooled replicate data.
Identifies Putative DE transcripts.
Fisher's exact tests (pooled replicates):
[Back-splice counts (BS) from transcript]
and [Total junctions from sample – BS]
Compare all time-points/conditions
Fisher's exact tests (pooled replicates):
[Back-splice counts (BS) from transcript]
and [Total Junctions from locus – B]
Compare all time-points/conditions
Multiple testing correction:
Benjamini-Hochberg FDR 0.05
Multiple testing correction:
Benjamini-Hochberg FDR 0.05
Analysis 2. Using numbers from
each replicate.
Controls for heterogeneity.
T-tests (replicates):
BS counts from transcript, normalised
by canonical junction count in replicate
Compare all time-points/conditions
T-tests (replicates):
BS counts from transcript, normalised
by total junction count from locus
Compare all time-points/conditions
Multiple testing correction:
Benjamini-Hochberg FDR 0.05
Multiple testing correction:
Benjamini-Hochberg FDR 0.05
Compare DE lists
DE transcripts,
controlling for sample and
parent locus changes

Figure S6

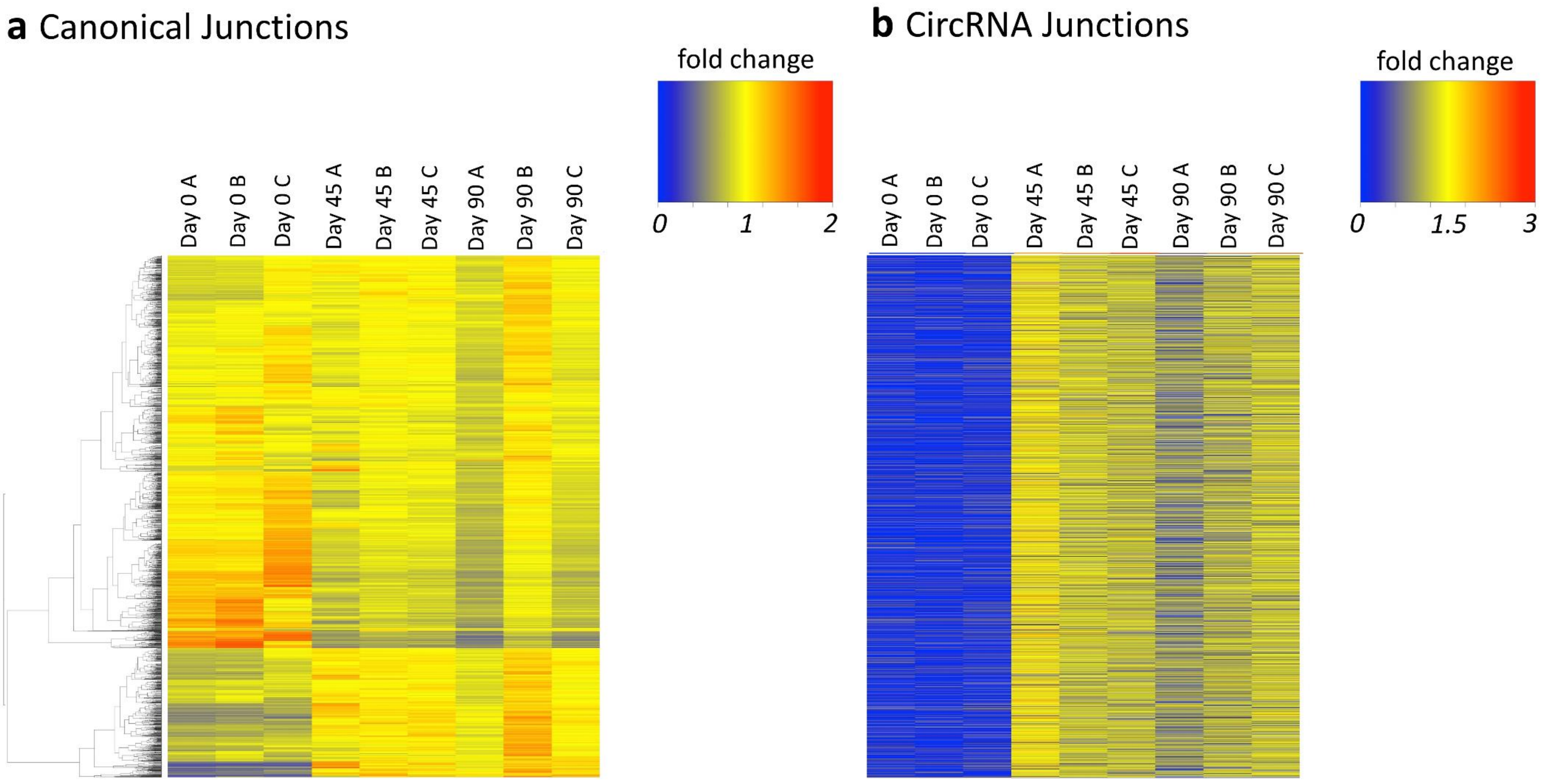
a Canonical Junctions
fold change
0
1
2
Day 0 A
Day 0 B
Day 0 C
Day 45 A
Day 45 B
Day 45 C
Day 90 A
Day 90 B
Day 90 C
b CircRNA Junctions
fold change
0
1.5
3
Day 0 A
Day 0 B
Day 0 C
Day 45 A
Day 45 B
Day 45 C
Day 90 A
Day 90 B
Day 90 C

Figure S7

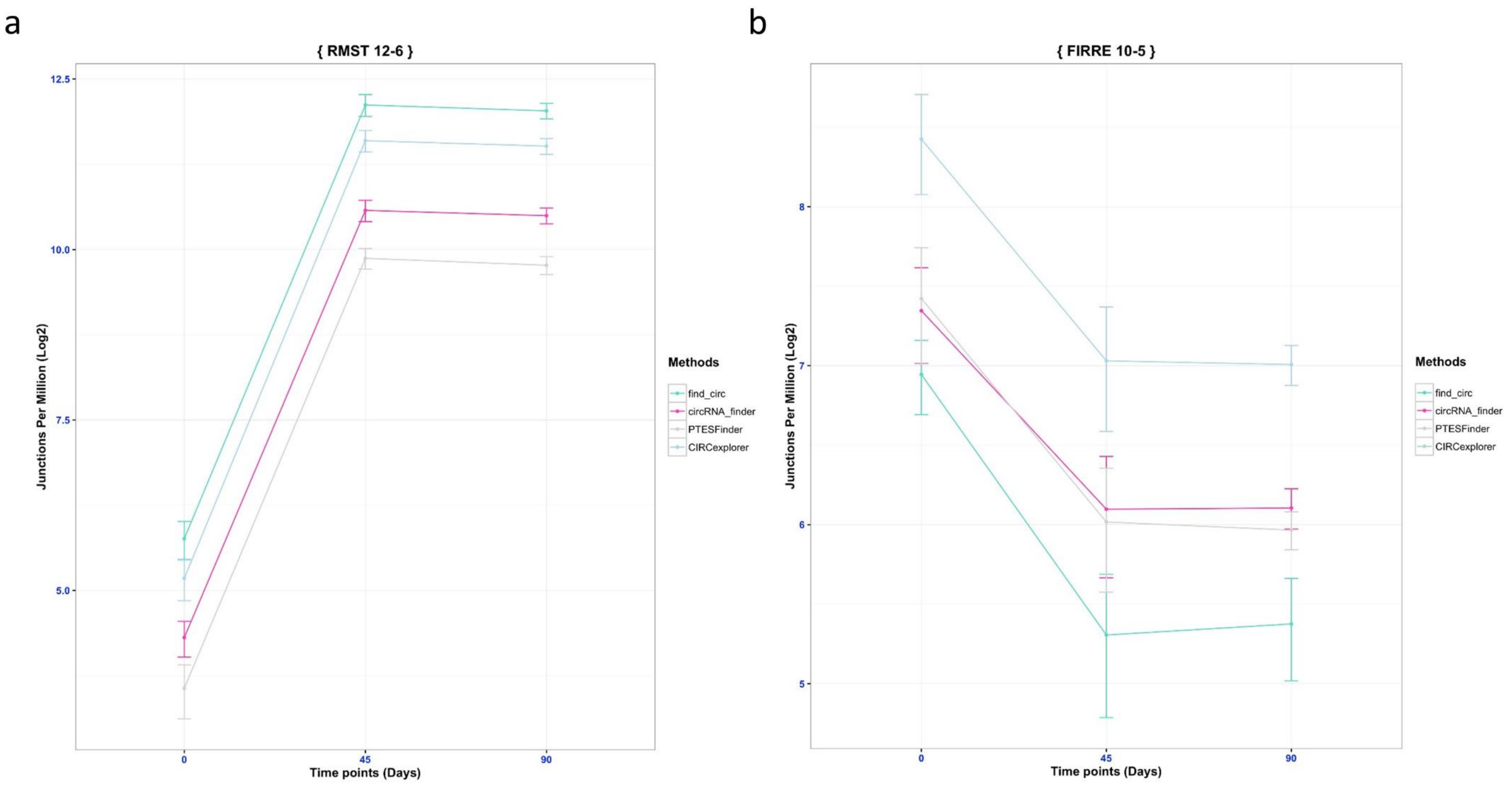

a
{ RMST 12-6 }
12.5
10.0
7.5
5.0
Junctions Per Million (Log2)
0
45
90
Time points (Days)
Methods
find_circ
circRNA_finder
PTESFinder
CIRCexplorer
b
{ FIRRE 10-5 }
8
7
6
5
Junctions Per Million (Log2)
0
45
90
Time points (Days)
Methods
find_circ
circRNA_finder
PTESFinder
CIRCexplorer

Figure S8

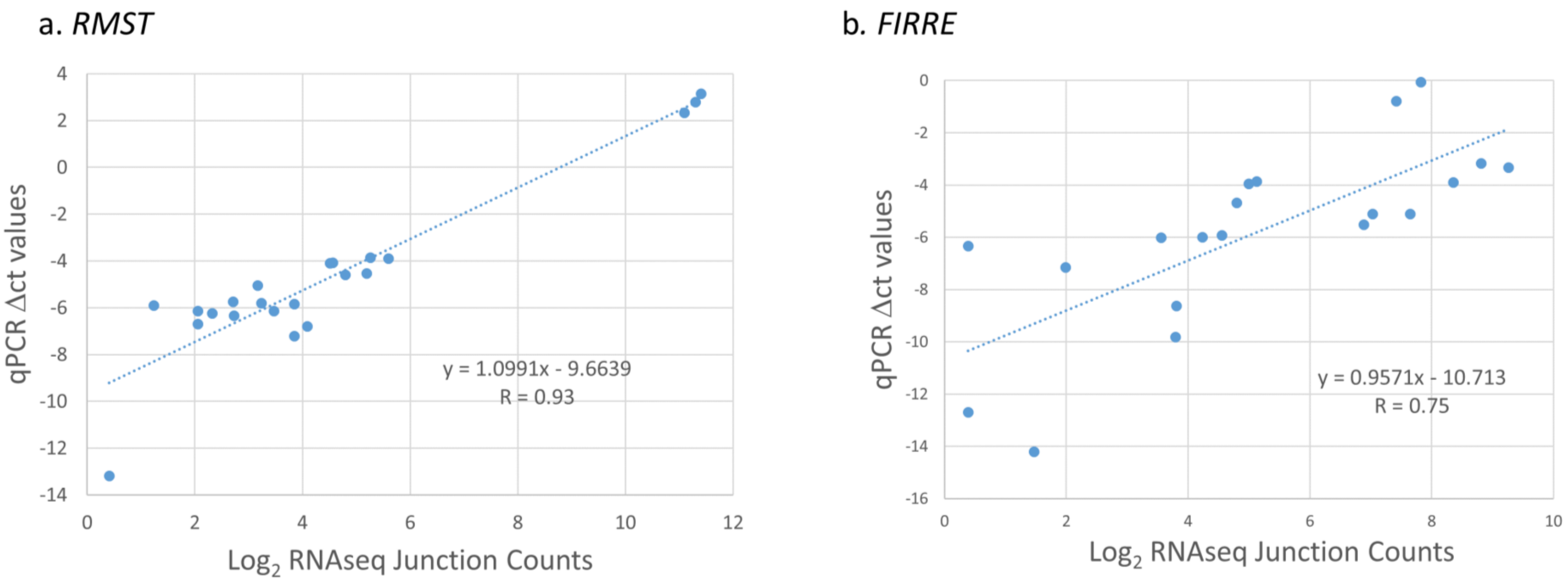

a. RMST
qPCR Δct values
Log2 RNAseq Junction Counts
y = 1.0991x - 9.6639
R = 0.93
b. FIRRE
qPCR Δct values
Log2 RNAseq Junction Counts
y = 0.9571x - 10.713
R = 0.75

Figure S9

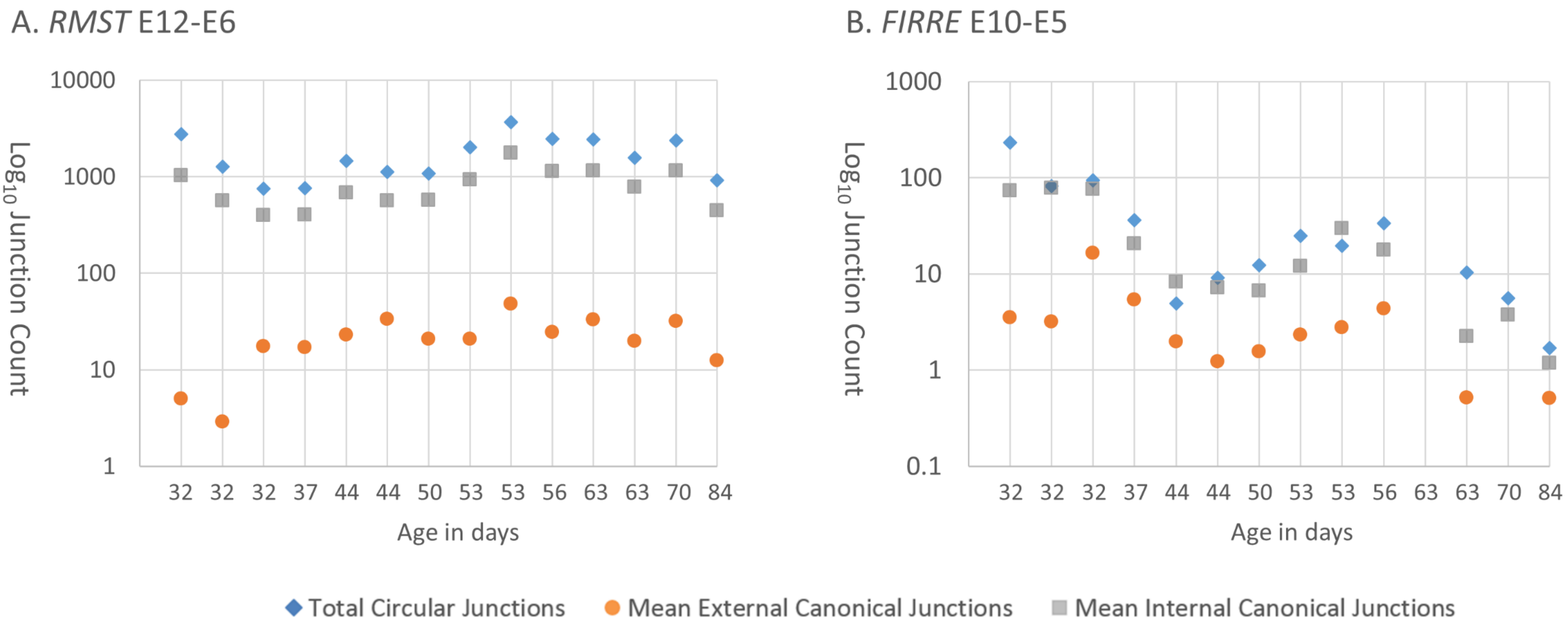
A. RMST E12-E6
B. FIRRE E10-E5
10000
1000
100
10
1
0.1
Log10 Junction Count
32 32 32 37 44 44 50 53 53 56 63 63 70 84
Age in days
Total Circular Junctions
Mean External Canonical Junctions
Mean Internal Canonical Junctions

Figure S10

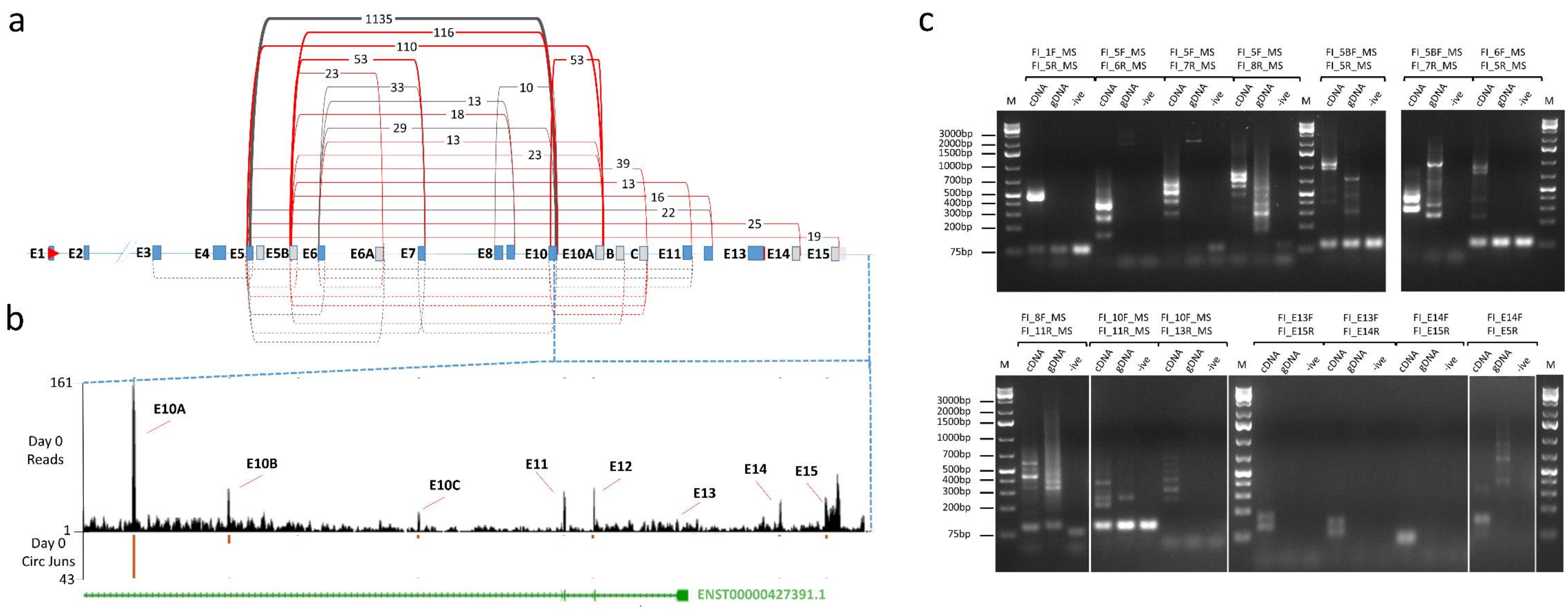

a
b
c
1135
116
110
53
53
E1
E2
E3
E4
E5
E5B
E6
E6A
E7
E8
E10
E10A
B
C
E11
E13
E14
E15
161
Day 0
Reads
1
Day 0
Circ Juns
43
E10A
E10B
E10C
E11
E12
E13
E14
E15
ENST00000427391.1
M
cDNA
gDNA
-ive
3000bp
2000bp
1500bp
1000bp
700bp
500bp
400bp
300bp
200bp
75bp
FI_1F_MS
FI_5R_MS
FI_5F_MS
FI_6R_MS
FI_5F_MS
FI_7R_MS
FI_5F_MS
FI_8R_MS
FI_5BF_MS
FI_5R_MS
FI_5BF_MS
FI_7R_MS
FI_6F_MS
FI_5R_MS
FI_8F_MS
FI_11R_MS
FI_10F_MS
FI_11R_MS
FI_10F_MS
FI_13R_MS
FI_E13F
FI_E15R
FI_E13F
FI_E14R
FI_E14F
FI_E15R
FI_E14F
FI_E5R

Figure S11

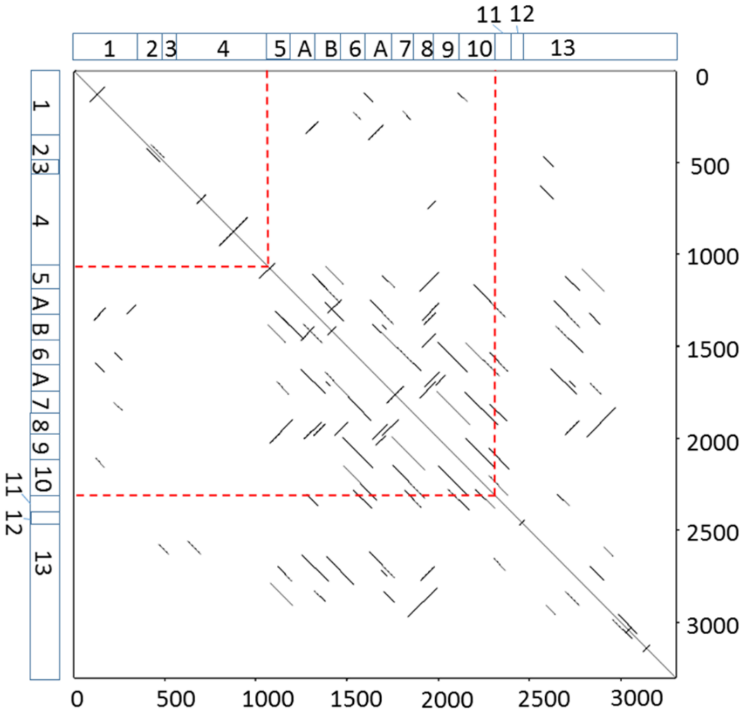
11 12
1 2 3 4 5 A B 6 A 7 8 9 10 13
0
500
1000
1500
2000
2500
3000
0 500 1000 1500 2000 2500 3000

Figure S12

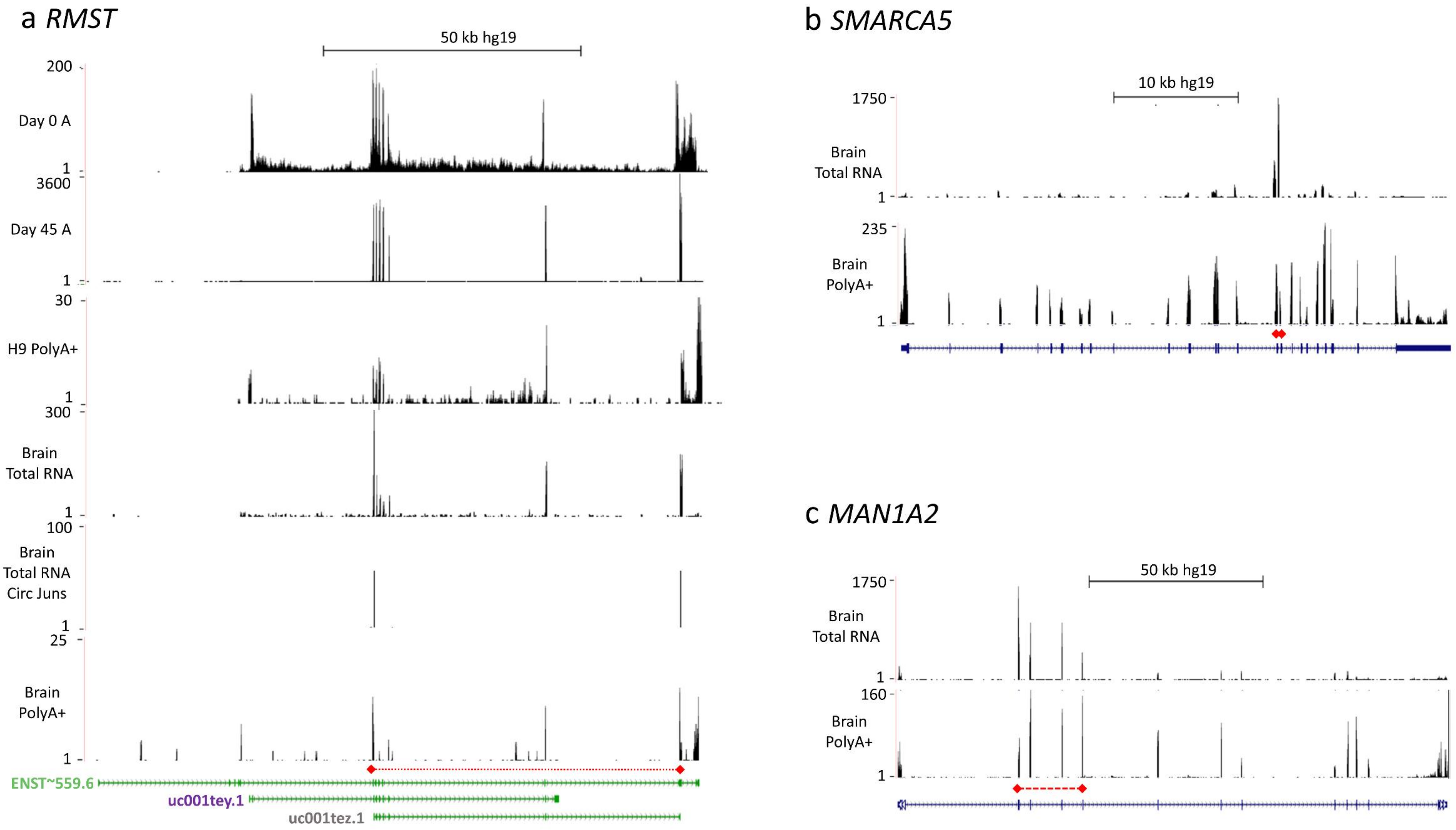

a RMST
50 kb hg19
200
Day 0 A
1
3600
Day 45 A
1
30
H9 PolyA+
1
300
Brain
Total RNA
1
100
Brain
Total RNA
Circ Juns
1
25
Brain
PolyA+
1
ENST~559.6
uc001tey.1
uc001tez.1
b SMARCA5
10 kb hg19
1750
Brain
Total RNA
1
235
Brain
PolyA+
1
c MAN1A2
50 kb hg19
1750
Brain
Total RNA
1
160
Brain
PolyA+
1

Supplementary References

1. Hyslop L, Stojkovic M, Armstrong L, Walter T, Stojkovic P, Przyborski S, et al. Downregulation of NANOG induces differentiation of human embryonic stem cells to extraembryonic lineages. Stem Cells. 2005;23(8):1035-43.
2. Capowski EE, Simonett JM, Clark EM, Wright LS, Howden SE, Wallace KA, et al. Loss of MITF expression during human embryonic stem cell differentiation disrupts retinal pigment epithelium development and optic vesicle cell proliferation. Hum Mol Genet. 2014;23(23):6332-44.
3. Naeem MA, Chavali VR, Ali S, Iqbal M, Riazuddin S, Khan SN, et al. GNAT1 associated with autosomal recessive congenital stationary night blindness. Investigative ophthalmology & visual science. 2012;53(3):1353-61.
4. Davidson AE, Millar ID, Urquhart JE, Burgess-Mullan R, Shweikh Y, Parry N, et al. Missense mutations in a retinal pigment epithelium protein, bestrophin-1, cause retinitis pigmentosa. American journal of human genetics. 2009;85(5):581-92.
5. Dizhoor AM, Ray S, Kumar S, Niemi G, Spencer M, Brolley D, et al. Recoverin: a calcium sensitive activator of retinal rod guanylate cyclase. Science. 1991;251(4996):915-8.
6. Kuehn MH, Hageman GS. Expression and characterization of the IPM 150 gene (IMPG1) product, a novel human photoreceptor cell-associated chondroitin-sulfate proteoglycan. Matrix Biol. 1999;18(5):509-18.
7. Conn SJ, Pillman KA, Toubia J, Conn VM, Salmanidis M, Phillips CA, et al. The RNA Binding Protein Quaking Regulates Formation of circRNAs. Cell. 2015;160(6):1125-34.
8. Lex A, Gehlenborg N, Strobelt H, Vuillemot R, Pfister H. UpSet: Visualization of Intersecting Sets. IEEE Trans Vis Comput Graph. 2014;20(12): 1983-92.
9. Izuogu OG, Alhasan AA, Alafghani HM, Santibanez-Koref M, Elliott DJ, Jackson MS. PTESFinder: a computational method to identify post-transcriptional exon shuffling (PTES) events. BMC Bioinformatics. 2016;17:31.
10. Memczak S, Jens M, Elefsinioti A, Torti F, Krueger J, Rybak A, et al. Circular RNAs are a large class of animal RNAs with regulatory potency. Nature. 2013;495:333-8.
11. Zhang X-O, Wang H-B, Zhang Y, Lu X, Chen L-L, Yang L. Complementary Sequence-Mediated Exon Circularization. Cell. 2014;159:134-47.
12. Westholm JO, Miura P, Olson S, Shenker S, Joseph B, Sanfilippo P, et al. Genome-wide analysis of drosophila circular RNAs reveals their structural and sequence properties and age-dependent neural accumulation. Cell Rep. 2014;9(5):1966-80.
13. Yang L, Duff MO, Graveley BR, Carmichael GG, Chen LL. Genomewide characterization of non-polyadenylated RNAs. Genome Biol. 2011;12(2):R16.
14. Brawand D, Soumillon M, Necsulea A, Julien P, Csardi G, Harrigan P, et al. The evolution of gene expression levels in mammalian organs. Nature. 2011;478(7369):343-8.
